# Rice LEAFY COTYLEDON1 Hinders Embryo Greening During the Seed Development

**DOI:** 10.3389/fpls.2022.887980

**Published:** 2022-05-10

**Authors:** Fu Guo, Peijing Zhang, Yan Wu, Guiwei Lian, Zhengfei Yang, Wu Liu, B. Buerte, Chun Zhou, Wenqian Zhang, Dandan Li, Ning Han, Zaikang Tong, Muyuan Zhu, Lin Xu, Ming Chen, Hongwu Bian

**Affiliations:** ^1^College of Life Sciences, Zhejiang University, Hangzhou, China; ^2^Hainan Institute, Zhejiang University, Yazhou Bay Science and Technology City, Sanya, China; ^3^Liangzhu Laboratory, Zhejiang University Medical Centre, Hangzhou, China; ^4^National Key Laboratory of Plant Molecular Genetics, CAS Centre for Excellence in Molecular Plant Sciences, Institute of Plant Physiology and Ecology, Chinese Academy of Sciences, Shanghai, China; ^5^College of Life Sciences, Shanghai Normal University, Shanghai, China; ^6^State Key Laboratory of Subtropical Silviculture, Zhejiang A&F University, Lin'an, China

**Keywords:** seed development, seed maturation, OsLEC1, photosynthesis, CRISPR/Cas9

## Abstract

LEAFY COTYLEDON1 (LEC1) is the central regulator of seed development in Arabidopsis, while its function in monocots is largely elusive. We generated *Oslec1* mutants using CRISPR/Cas9 technology. *Oslec1* mutant seeds lost desiccation tolerance and triggered embryo greening at the early development stage. Transcriptome analysis demonstrated that *Oslec1* mutation altered diverse hormonal pathways and stress response in seed maturation, and promoted a series of photosynthesis-related genes. Further, genome-wide identification of OsLEC1-binding sites demonstrated that OsLEC1 bound to genes involved in photosynthesis, photomorphogenesis, as well as abscisic acid (ABA) and gibberellin (GA) pathways, involved in seed maturation. We illustrated an OsLEC1-regulating gene network during seed development, including the interconnection between photosynthesis and ABA/GA biosynthesis/signaling. Our findings suggested that OsLEC1 acts as not only a central regulator of seed maturation but also an inhibitor of embryo greening during rice seed development. This study would provide new understanding for the OsLEC1 regulatory mechanisms on photosynthesis in the monocot seed development.

## Introduction

Seeds store the genetic hardware within the embryo, which guarantees the orderly unfolding of the plant's next life cycle in interaction with the environment (Sreenivasulu and Wobus, 2013). During seed development, the major tissue types and stem-cell niches of plants are established. Thus, knowledge of seed development is essential for a full understanding of plant development (Gillmor et al., 2020). Plant seed development is divided into two phases: morphogenesis phase and maturation phase. During the morphogenesis phase, the basic body plan of the embryo and endosperm are established (Lau et al., 2012; Li and Berger, 2012); chloroplast biogenesis and photosynthesis are also initiated during this period in many angiosperm taxa (Puthur et al., 2013). During the maturation phase, photosynthesis, the accumulation of storage compounds, and the induction of desiccation tolerance and preparation for seed dormancy are the main processes (Jo et al., 2019).

Seed development is genetically controlled by at least four regulators, LEAFY COTYLEDON 1 (LEC1), LEC2, FUSCA 3 (FUS3), and ABSCISIC ACID INSENSITIVE 3 (ABI3) (Giraudat et al., 1992; Meinke, 1992; Luerssen et al., 1998; Stone et al., 2001). Generally, *lec1, lec2, fus3*, and *abi3* mutants represent reduction of desiccation tolerance and seed dormancy (Meinke, 1992; Keith et al., 1994; Meinke et al., 1994; West et al., 1994; Parcy et al., 1997; Lotan et al., 1998; Nambara et al., 2000; Stone et al., 2001; Pelletier et al., 2017). LEC1 acts as a central regulator of seed development through combinatorial binding with ABA-RESPONSIVE ELEMENT-BINDING PROTEIN 3 (AREB3), BASIC LEUCINE ZIPPER 67 (bZIP67), and ABI3 (Jo et al., 2019).

In Arabidopsis, loss-of-function mutations of *LEC1* cause defects in storage proteins and lipid accumulation, acquisition of desiccation tolerance, and suppression of germination and leaf primordia initiation (Meinke, 1992; Meinke et al., 1994; West et al., 1994; Santos-Mendoza et al., 2008). Moreover, ectopic expression of *LEC1* induces embryonic development and the activation of genes involved in maturation and storage, as well as lipid accumulation in vegetative organs (Lotan et al., 1998; Kagaya et al., 2005; Mu et al., 2008). A few of studies have explored the effects of the overexpression of *LEC1* in carrot, maize, and rice. Expressing carrot *C-LEC1* driven by the Arabidopsis *LEC1* promoter could complement the viviparous and desiccation intolerant defects of Arabidopsis *lec1-1* mutant (Yazawa et al., 2004). Overexpression of maize (*Zea mays*) *LEAFY COTYLEDON 1* (*ZmLEC1)* increases seed oil production by up to 48% but reduces seed germination and leaf growth (Shen et al., 2010). In rice, *OsLEC1* overexpression results in abnormalities in the development of leaves, panicles, and spikelets (Zhang and Xue, 2013). Heterologous expression of *OsNF-YB7* (*OsLEC1*) in Arabidopsis *lec1-1* complements the *lec1-1* defects. OsNF-YB7 defect causes lethality (Niu et al., 2021). However, how LEC1 regulates rice seed development is not clear.

In the present study, we generated *Oslec1* mutants using a gene-editing technique. Phenotype analysis revealed that dry seeds of *Oslec1* mutants could not germinate but fresh seeds of the mutants germinated normally, suggesting lack of desiccation tolerance. Notably, embryos of mutants turned green during early seed development. Subsequently, RNA-seq transcriptional profiling and ChIP-seq were used to identify the underlying mechanisms via which *OsLEC1* regulates photosynthesis-related genes and seed-maturation related genes. Our studies provide new insights that OsLEC1 is an inhibitor of embryo greening during rice seed development, in addition to being a central regulator of seed maturation.

## Results

### *OsLEC1* Deficiency Disrupts Seed Desiccation Tolerance and Germination

**Figure 1:**
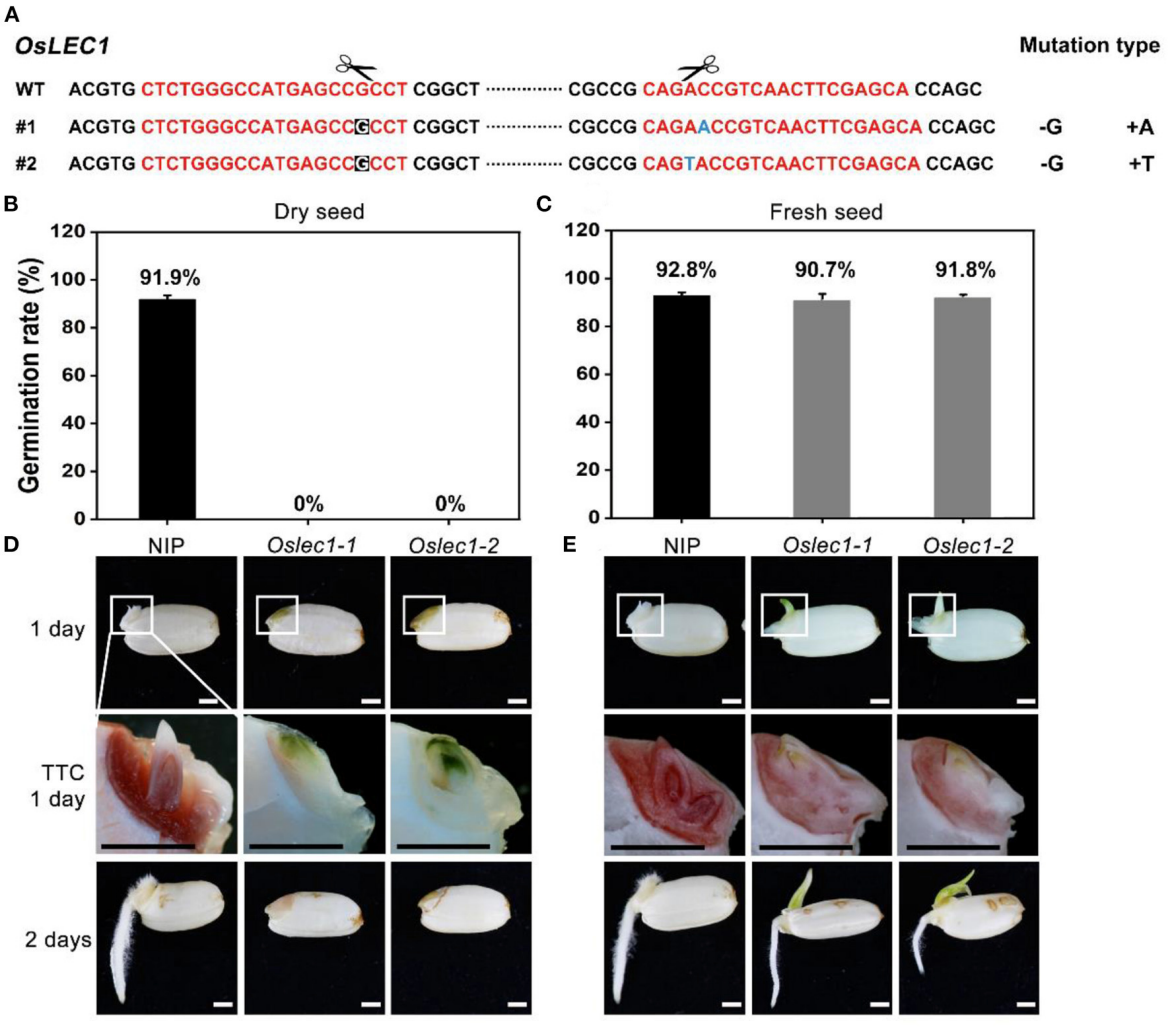
Germination phenotypes of *Oslec1* mutants. **(A)** Mutation sites in the *Oslec1* mutant DNA. The red sequence represents the designed guide RNA (gRNA) for the wild type rice and the scissors represent the predicted knockout sites. The letters with a black background represent the deleted bases, and the blue letter represents the inserted base. **(B,C)** Germination rates of dry seeds and the fresh seeds of the wild type and *Oslec1* mutants after 1 day of imbibition at 37°C. **(D,E)** Photos of the wild type and *Oslec1* mutant seeds after 1 day of imbibition at 37°C (1 day) and 1 day on wet filter paper at 28°C (2 days). Photos of group “TTC 1 day” were the amplification of the regions in white boxes in group “1 day,” indicating the embryos stained by TTC for 3 h at 37°C. Scale bars = 1 mm.

We first established that LEC1 was largely conserved while typically different in a few sites between monocots and dicots by performing multiple alignments (Supplementary Figures 1A,B). To determine the physiological functions of OsLEC1 in rice, we used the CRISPR/Cas9 technology to knock out *OsLEC1*. We generated more than 30 transformant plants in the T0 generation, in which seven homozygous deletion mutants were screened using PCR and DNA sequencing (data not shown). DNA sequencing analysis showed that *Oslec1-1* and *Oslec1-2* contained a 1 bp deletion (G) at the gRNA1 site, and a 1 bp insertion (A or T) at the gRNA2 site (Figure 1A), leading to frameshift mutations and generating premature stop codons at aa157 (Supplementary Figure 1C). Thus, *Oslec1-1* and *Oslec1-2* mutations disrupted the original protein structure from 254 to 157 aa. Except for the shorter height of the mutants compared to the wild type plants, we did not observe obviously abnormal morphological phenotypes, including the tillering or grain number (Supplementary Figure 2). While >90% of the wild type seeds germinated after drying at 37°C for 3 days, none of the mutant seeds germinated (Figures 1B,D). Surprisingly, freshly harvested mutant seeds germinated and even rooted at 24 h after imbibition, when the wild type plant had not germinated yet (Figure 1E, Supplementary Figure 3). Finally, >90% of *Oslec1-1* and *Oslec1-2* fresh seeds germinated, similar to the wild type (Figure 1C). Further, 2,3,5-Triphenyltetrazolium chloride (TTC) staining revealed that the dry seeds of *Oslec1* mutants displayed green primordium without any red tissues, indicating completely lethal embryos in the dry mutant seeds (Figure 1D). Interestingly, green leaf primordia were observed in *Oslec1-1* and *Oslec1-2* embryos but not in wild type ones, suggesting chlorophyll accumulation in the *Oslec1* embryos. Meanwhile, we tested the embryo vitality of freshly harvested seeds (about 25 days after pollination [DAP]) using TTC staining. The embryo vitality of *Oslec1* fresh seeds was similar to that of the wild type seeds (Figure 1E).

Our results revealed that the *Oslec1* mutation disrupted desiccation tolerance but initiated chlorophyll accumulation in seed development, suggesting that *OsLEC1* acts as a key regulator of seed maturation in rice.

### *OsLEC1* Deficiency Leads to a Green Embryo During Seed Development

**Figure 2:**
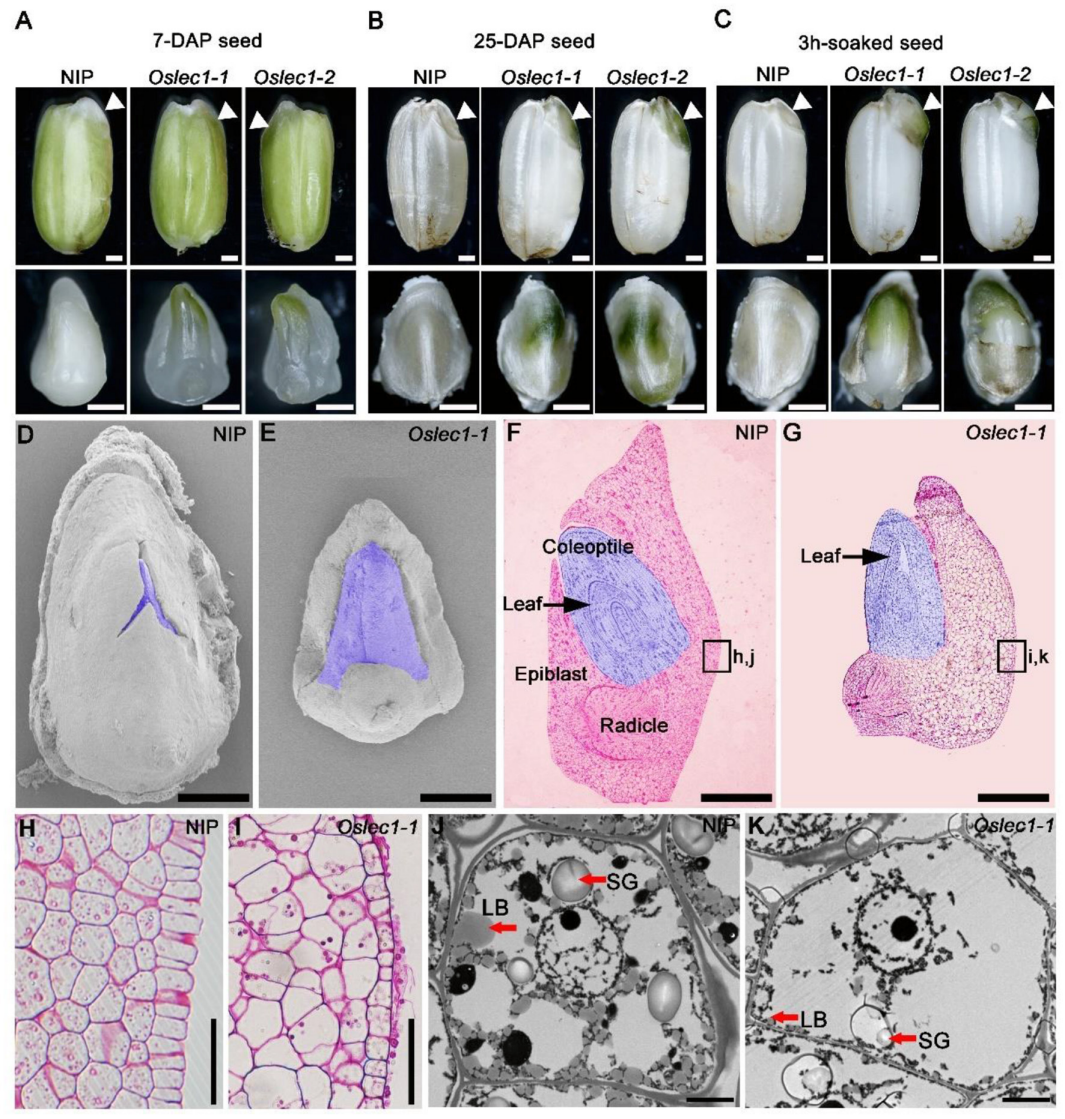
Phenotypes of the developing seeds of *Oslec1* mutants. **(A)** Seeds and embryos (7-DAG) of wild type plants and *Oslec1* mutants. **(B)** Seeds and embryos (25-DAP) of wild type plants and *Oslec1* mutants. **(C)** Seeds and embryos of wild type plants and *Oslec1* mutants soaked in water for 3 h at 37°C. Scale bars = 500 μm. White triangles indicate the embryonic regions in complete seeds. **(D,E)** Scanning electron micrographs of embryos (7-DAP) of wild type plants and *Oslec1* mutants. The purple-colored regions indicate the germs of wild type plants and *Oslec1* mutants. Bars = 500 μm. **(F,G)** Longitudinal resin sections of embryos (7-DAP) of wild type plants and *Oslec1* mutants. The purple-colored regions indicate the germs of wild type plants and *Oslec1* mutants. Black arrows indicate the leaves in the coleoptiles. The leaves of *Oslec1* mutants are stretched out, and the epiblast wrapping the coleoptile is missing. Scale bars = 500 μm. **(H,I)** Amplification of the regions in black boxes in F and G, showing the difference in scutella parenchyma between the wild type and *Oslec1-1* mutant. Scale bars = 50 μm. **(J,K)** Electron micrographs of wild type and *Oslec1-1* embryos. Wild type **(J)** and *Oslec1-1* **(K)** scutellum sections from 7-DAP embryos. Scale bars = 2 μm.

Further, we examined the embryo phenotypes of the *Oslec1* mutants and wild type plants at different stages of rice seed development. Wild type embryos remain white during the whole process of rice seed development. In contrast to the wild type embryos, *Oslec1* mutant embryos displayed green apical shoots at 7 DAP (Figure 2A, Supplementary Figure 4). Subsequently, the apical shoot remained green in *Oslec1* mutant embryos at 25 DAP (Figure 2B, Supplementary Figure 4). Surprisingly, after imbibing water for only 3 h at 37°C, the epiblasts covering the embryos were split by the sprouting shoots in the *Oslec1* mutants, indicating more rapid germination in comparison to the wild type plants (Figure 2C, Supplementary Figure 4).

Scanning electron microscopy (SEM) showed that at 7 DAP, the shoot apical meristems in wild type embryos were embedded in the covering epiblast (Figure 2D), while those in the mutants were exposed (Figure 2E). Semi-thin longitudinal sections of embryos exhibited an ordered three-layer leaf primordium in the intact coleoptile of wild type embryos (Figure 2F), whereas in *Oslec1* mutant embryos at 7 DAP, the primary leaves were spread out (Figure 2G). Furthermore, the wild type embryos were bent while *Oslec1* mutant embryos were straight (Figures 2F,G). Thus, the green embryos of *Oslec1* mutants showed that leaf primordia initiation were triggered in the embryo. Moreover, the scutella parenchyma of *Oslec1* mutants was abnormal compared with those of the wild type plants (Figures 2H,J). Transmission electron microscopy (TEM) revealed many lipid bodies and large starch grains in wild type embryos at 7 DAP, while major storage products were almost absent in *Oslec1* mutant embryos (Figures 2J,K). The above data suggested that *OsLEC1* might play a crucial role in the regulation of germination inhibition, and accumulation of storage compounds in rice seed development.

### *OsLEC1* Is Expressed Predominantly in Immature Embryos During Early Seed Development

We analyzed the expression pattern of *OsLEC1* based on an expression database (http://expression.ic4r.org/) (Xia et al., 2017) (Supplementary Figure 5A). The results showed that *OsLEC1* was highly expressed in the callus and immature seeds. Results of qRT-PCR further indicated that mRNA of OsLEC1 accumulated in the embryos but not in endosperm or glume of 20-DAP seeds (Supplementary Figure 5B). Subcellular localization showed that OsLEC1-GFP was enriched in the nucleus, which was indicated by OsIAA1-mCherry in rice protoplasts (Supplementary Figure 5C).

**Figure 3:**
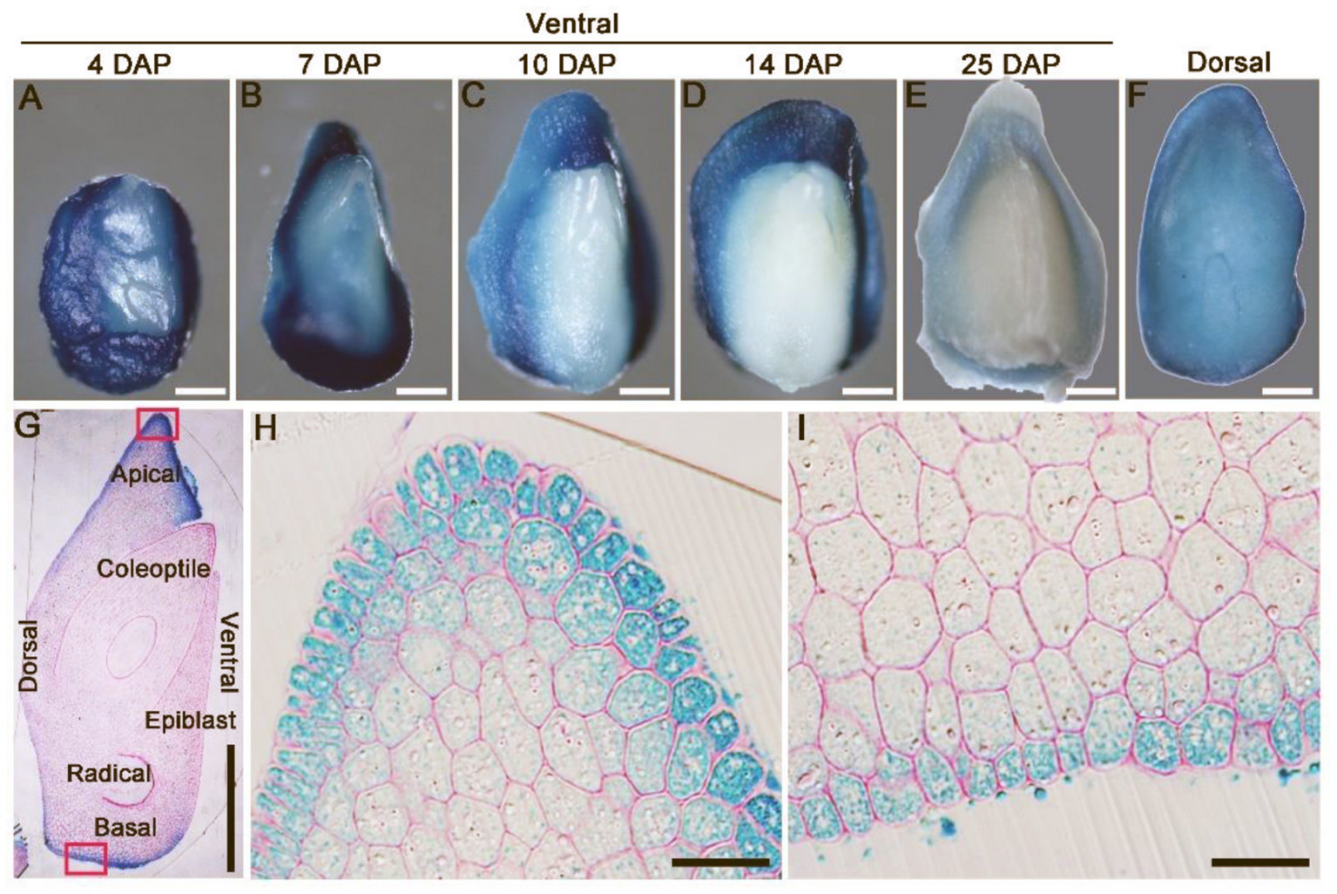
Expression pattern of *OsLEC1*. **(A–E)** Ventral side of *pOsLEC1:GUS* embryos (4–25 DAP) stained in X-Gluc solution for 3 h at 37°C. Scale bars = 200 μm. **(F)** Dorsal side of *pOsLEC1:GUS* embryos (14 DAP) stained in X-Gluc solution for 3 h at 37°C. Scale bars = 200 μm. **(G)** Longitudinal resin section of embryos (7 DAP) of *pOsLEC1:GUS* embryo stained in X-Gluc solution overnight at 37°C. Scale bars = 200 μm. **(H,I)** Amplification of the regions in black boxes in G, showing the apical **(H)** and basal **(I)** parts of the scutella parenchyma, respectively. Scale bars = 50 μm.

*pOsLEC1:GUS* was expressed strongly in the dorsal section of immature embryos within 4-7 DAP (Figures 3A,B) and then declined to a relatively low level at 25 DAP (Figures 3C–F). Further, semi-thin sections of resin-embedded embryos showed that *OsLEC1* was predominantly expressed in the cells of scutella parenchyma, especially in the apical and basal part of the embryo at 8 DAP (Figures 3G–I). GUS activity could be detected at the scutellum of germinating mature seeds and in callus-induced from scutellum, but not in leaves and roots of seedlings within 14 DAG (Supplementary Figure 6). Expression patterns of *OsLEC1* indicated its possible function in the early stage of rice embryo development.

### *Oslec1* Mutation Influences Multiple Developmental Processes in Rice Seeds

**Figure 4:**
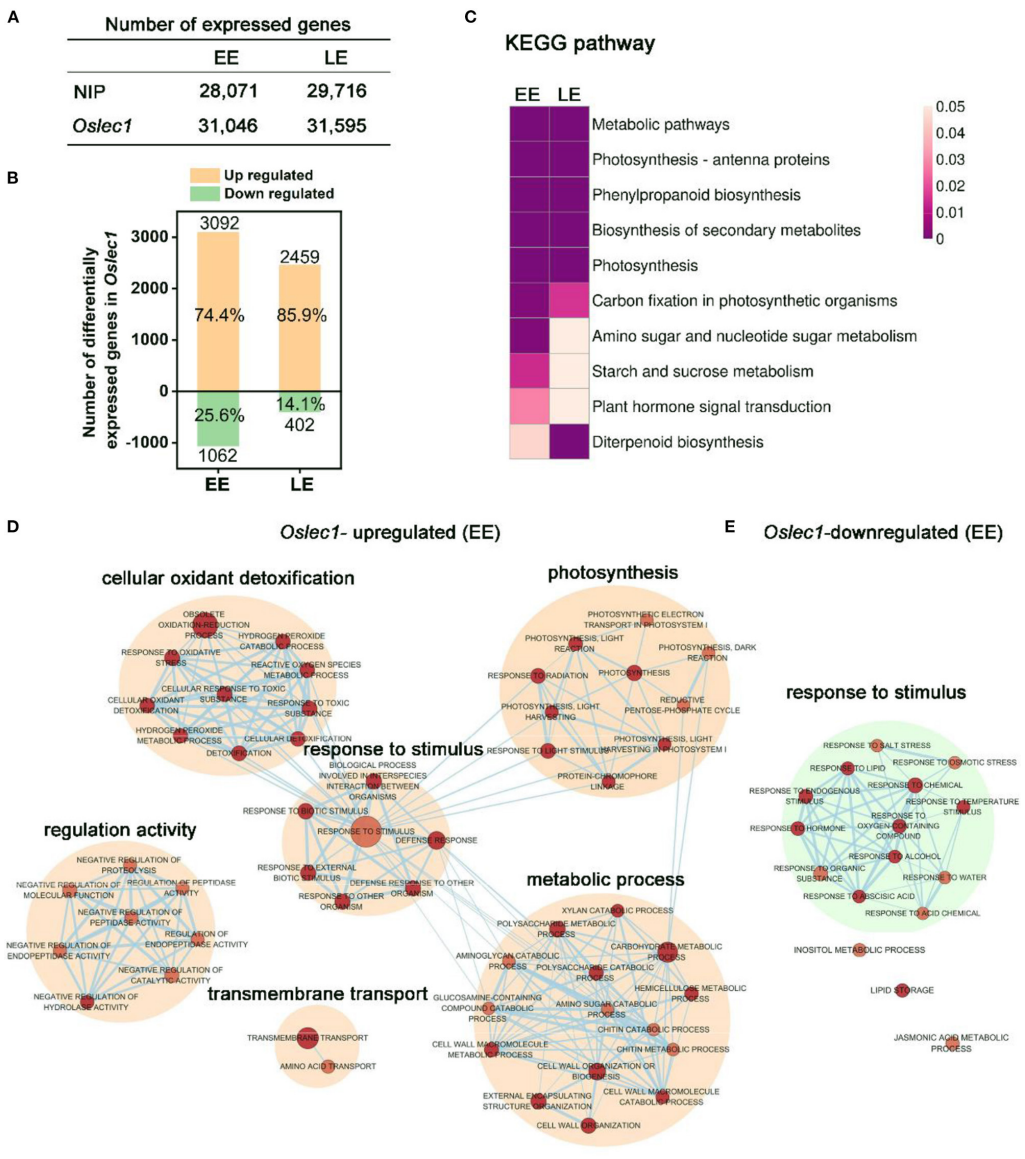
mRNA profiling of *Oslec1* mutant embryos at early (EE) and late (LE) development stages. **(A)** The number of differentially expressed genes detected in wild type and *Oslec1-1* embryos at EE (7 DAP) and LE (25 DAP) stages. **(B)** The number of upregulated and downregulated genes in *Oslec1-1* embryos compared with that in wild type plants. **(C)** KEGG pathway analysis of differentially expressed genes detected in wild type and *Oslec1-1* embryos at EE and LE stages. **(D,E)** GO term analysis of upregulated **(D)** and downregulated **(E)** genes in *Oslec1-1* embryos compared with those in the wild type ones.

To identify the genes that were regulated by *OsLEC1*, we performed RNA-seq in *Oslec1-1* mutant and wild type embryos at two different stages of seed development: the early-stage (EE) embryos at 7 DAP and late-stage (LE) embryos at 25 DAP, representing the morphogenesis and maturation phases, respectively (Figure 4).

Spearman correlation of 12 samples in the two stages showed good repeatability with a coefficient (*R*^2^) of above 0.99, and good heterogeneity with an *R*^2^ below 0.75 (Supplementary Figure 7). The number of genes detected in wild type and *Oslec1* mutant embryos at the two stages are shown in Figure 4A. Compared with the wild type, there were 3,092 and 2,459 upregulated genes [*p* < 0.05, log_2_ (Fold change)≥2] in *Oslec1-20* mutants at the two stages (EE and LE embryos), while there were only 1,062 and 402 downregulated genes [*p* < 0.05, log_2_ (Fold change) ≤ −2], respectively (Figure 4B and Supplementary Tables 1–4). The percentage of *Oslec1-*upregulated genes among the total number of differentially expressed genes (DEGs) (74.4 and 85.9%) was more than that of downregulated genes (25.6 and 14.1%), suggesting that *OsLEC1* mutation activated more genes than it suppressed. Thus, OsLEC1 might function mainly as a repressor in rice seed development.

Kyoto Encyclopedia of Genes and Genomes (KEGG) analysis showed that top pathways (*p* < 0.05) of differentially expressed genes detected in wild type and *Oslec1-1* embryos were similar at EE and LE stages (Figure 4C), including metabolic pathways, photosynthesis-antenna proteins, phenylpropanoid biosynthesis, biosynthesis of secondary metabolites, photosynthesis, and carbon fixation in photosynthetic organisms. KEGG terms related to amino sugar and nucleotide sugar metabolism, starch and sucrose metabolism, and plant hormone signal transduction were selectively enriched at the EE stage, while diterpenoid biosynthesis was preferentially enriched at the LE stage, as shown in Figure 4C and Supplementary Table 5. The results suggested that *Oslec1* mutation affects many biological pathways involved in photosynthesis, biomacromolecule biosynthesis and metabolism, and hormone signal transduction in seed development.

To further explore the transcriptional regulation of *OsLEC1* in the seed development, we identified all the GO terms significantly enriched (*p* value <0.01) in the biological progress of the two stages (Figure 4D, Supplementary Tables 6–9). The network showed that *Oslec1*-upregulated genes were mainly enriched in GO terms involved in photosynthesis, cellular oxidant detoxification, response to stimulus, metabolic process and regulation activity, while *Oslec1*-downregulated genes were mainly enriched in GO terms involved in response to stimulus biological process at EE and LE stages (Figure 4D, Supplementary Figure 8). The GO terms for *Oslec1*-upregulated mRNAs related to photosynthesis include photosynthesis, light harvesting, and light reactions, consistent with the embryo greening phenotype of *Oslec1* mutants (Figures 2, 4D). The GO terms for *Oslec1*-downregulated mRNAs involved in response to stimulus include response to water, ABA, hormones, and temperature stimulus (Figure 4E), consistent with the desiccation intolerant and rapid germination phenotypes related to seed dormancy (Figures 1, 2). Together, RNA-seq analysis suggested that *Oslec1* mutation affects two aspects in rice seed development: promoting photosynthesis-related pathways and interrupting seed maturation process.

### *Oslec1* Mutation Upregulates a Series of Genes Involved in Photosynthesis and Photomorphogenesis

**Figure 5:**
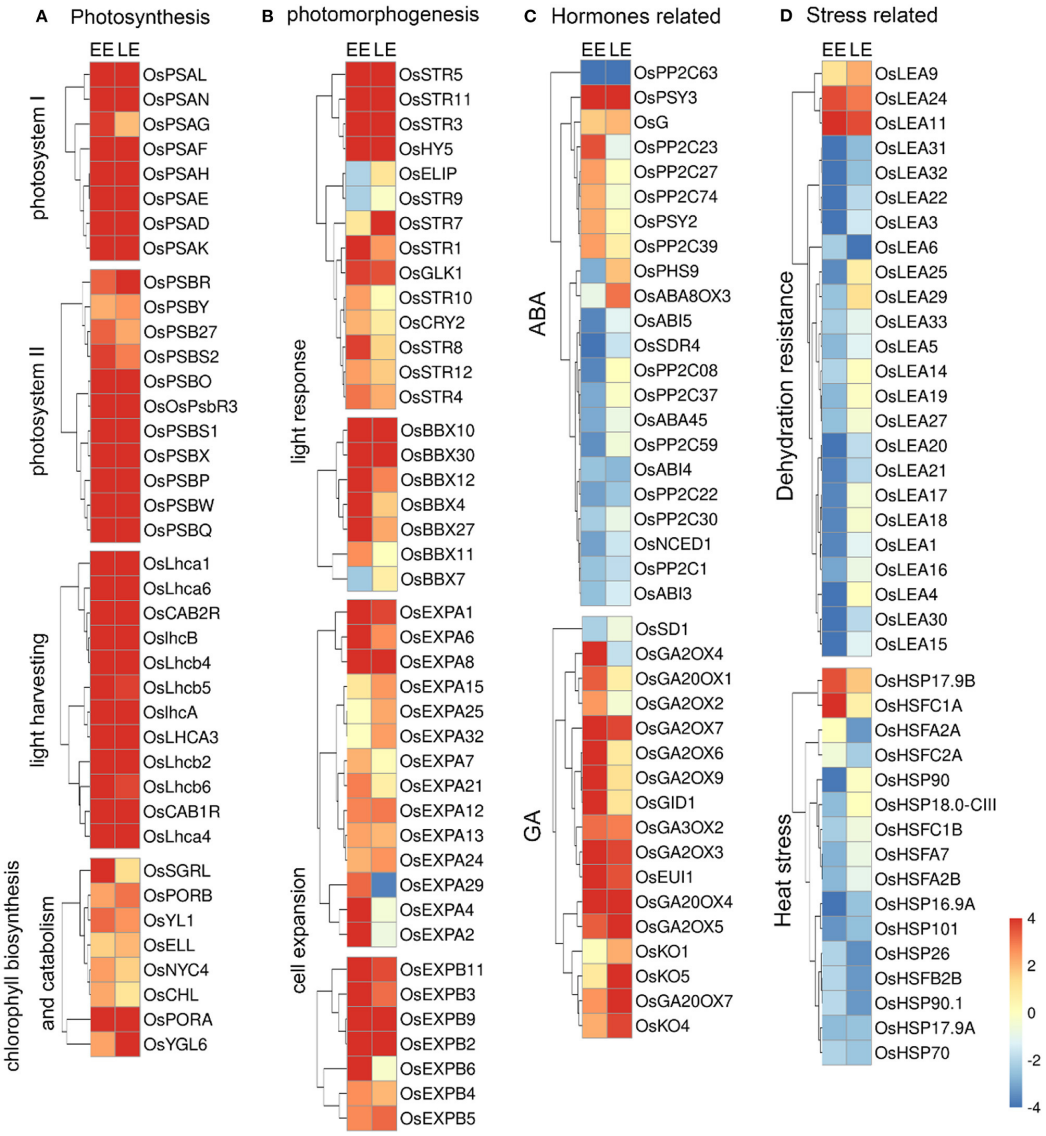
Heat maps of differentially expressed genes in *Oslec1* embryos. **(A)** Heat maps of genes involved in photosynthesis, **(B)** photomorphogenesis, **(C)** hormone pathways, and **(D)** stress responses, including dehydration resistance and heat stress response.

We designated genes regulated by *OsLEC1* as those whose expression was at least four-fold higher or lower [log2(Foldchange)≥2 or < −2] in *Oslec1* mutants than that in wild type seeds at the same stage at a statistically significant level (*p* < 0.05). Compared with wild type embryos, most genes involved in photosystem I (*OsPSA*s), photosystem II (*OsPSB*s), and light-harvesting complex (*OsLHCAs* and *OsLHCBs*) (Ben-Shem et al., 2003; Gao et al., 2018) were synergistically upregulated in *Oslec1-1* embryos (Figure 5A). In addition, several DEGs involved in chloroplast development, as well as chlorophyll biosynthesis and degradation (Bollivar, 2006; Hu et al., 2021) were also upregulated, such as *OsSGRL, OsPORA, OsPORB*, and *OsNYC4* (Figure 5A). Chloroplast biosynthesis genes are in the majority, and the *OsPORA* with the largest increase is chloroplast biosynthesis related. The results suggested that *OsLEC1* repressed the transcription of many genes related to photosynthesis during rice embryo development.

In addition, DEGs involved in photomorphogenesis, and light response were analyzed (Figure 5B). The results showed that *OsCRY2, OsHY5, OsBBX (4, 7, 10, 11, 12, 27, 30), OsEXPA (1, 2, 4, 6, 7, 8, 12, 13, 15, 21, 24, 25, 29, 32)*, and *OsEXPB (2, 3, 4, 5, 6, 9*,*11)* were significantly upregulated at least in one stage in the *Oslec1* embryos (Figure 5B, Supplementary Table 10). Ten members of the sulfurtransferase (Str) family (Bartels et al., 2007), were upregulated in the *Oslec1* mutant at least in one stage, including *OsStr (1, 3*, 4, *5*, 7-*12)*. In addition, *OsGLK1* (*GOLDEN2-LIKE1*) (Waters et al., 2009), was upregulated significantly at the EE and LE stages.

The above results demonstrated that *Oslec1* mutation upregulated a series of genes involved in photosynthesis and photomorphogenesis in the developing embryos.

### *Oslec1* Mutation Regulates Genes Involved in Diverse Hormonal Pathways and Stress Response

We identified the transcription of genes involved in hormone biosynthesis, signaling, and accumulation of six main hormones: abscisic acid (ABA), gibberellin (GA), brassinosteroid (BR), zeatin, ethylene, and auxin (De Paepe and Van der Straeten, 2005; Yamaguchi, 2008; Dong et al., 2015; Keshishian and Rashotte, 2015; Kim and Russinova, 2020; Tan et al., 2021) (Figure 5C, Supplementary Figure 10). *OsPP2C (1, 08, 22, 30, 37, 59, 63), OsNCED1, OsSDR4*, and *OsABA45* (involved in ABA biosynthesis), as well as *OsABI3, OsABI4*, and *OsABI5* (involved in ABA signaling) were significantly downregulated in the *Oslec1-1* mutant. Conversely, *OsKO (1, 4, 5), OsGA2OX (2, 3, 4, 5, 6, 7, 9), OsGA3OX2, OsGA20OX (1, 4, 7)*, and *OsGID1* (involved in GA biosynthesis) were significantly upregulated in the mutant (Figure 5C). In other hormonal pathways, several genes were differentially regulated. *OsCYP735A3* in zeatin biosynthesis and *OsHK2* in zeatin signaling, *OsBU1, OsSEPK1*, and *OsLAC15* in BR response, *OsACO (1, 2, 5)* in ethylene biosynthesis and *OsETR4* in ethylene signaling, *OsYUCCA (2, 6)* in auxin biosynthesis, *OsGH3-13* in auxin accumulation, *OsIAA (2, 12, 16)* and many *OsSAURs* in the auxin signaling pathway were significantly upregulated (Supplementary Figure 9). Overall, the majority of the genes related to GA, zeatin, BR, ethylene, and auxin pathways were activated in the *Oslec1-1* embryos. In contrast, *Oslec1* mutation suppressed ABA biosynthesis and signaling in the developing seeds.

Stress response-related genes including *OsLEA*s, *OsHSP*s, and *OsHSF*s were also significantly dysregulated in the *Oslec1-1* mutant (Figure 5D, Supplementary Table 11). Results showed that 21 *OsLEA*s were significantly downregulated, while only three were upregulated, such as *OsLEA9, OsLEA11*, and *OsLEA24*. Similarly, most *OsHSP* and *OsHSF* genes were downregulated in the *Oslec1-1* mutant. It is noteworthy that the expression levels of *OsLEA* genes were more significantly downregulated at the EE stage than the LE stage (Figure 5D), suggesting that *Oslec1* mutation affected expression of *OsLEA*s predominantly in the early stage of seed development.

Expression levels of nine representative genes were detected in EE and LE stage embryos using qRT-PCR. The results showed that *OsPSAN, OsPSAW, OsLHCA3, OsPORB, OsGA2ox3*, and *OsGA2ox9* were significantly upregulated in the *Oslec1* mutant, while *OsABI3, OsLEA1* and *OsLEA17* were significantly downregulated in early and late stages of seed development, consistent with the RNA-seq results (Supplementary Figure 10).

Overall, the transcriptome analysis of the *Oslec1* mutant suggested that OsLEC1 regulated diverse sets of genes involved in photosynthesis, photomorphogenesis, stress response, and diverse hormones pathways in seed development.

### Genome-Wide Identification of OsLEC1-Binding Sites

**Figure 6:**
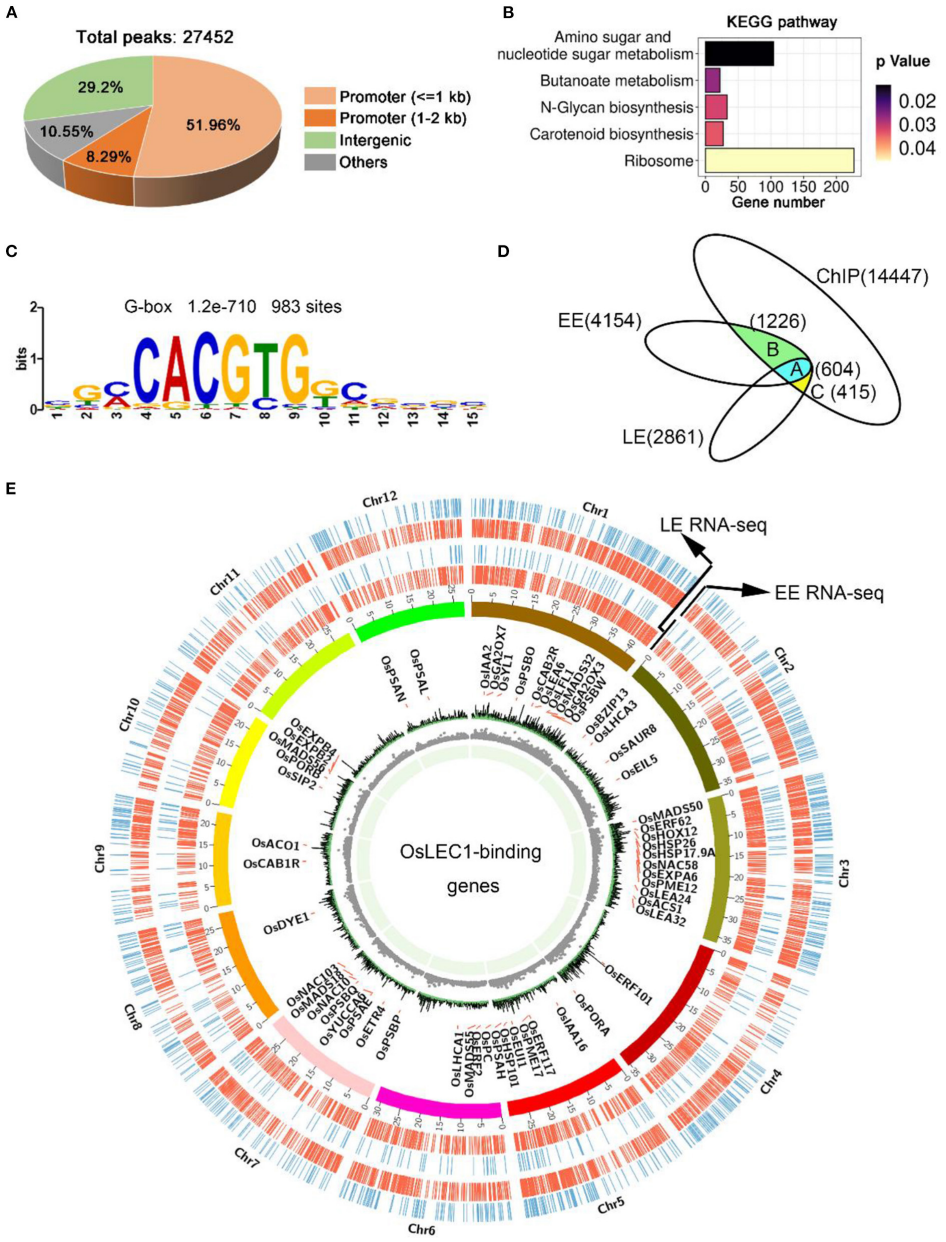
OsLEC1-binding genes detected using ChIP-seq. **(A)** The distribution of peaks on the rice genome according to ChIP-seq results. **(B)** KEGG pathway analysis of OsLEC1-binding genes. **(C)** DNA sequence motif G-box that enriched in LEC1-bound genomic regions 1 kb upstream of target genes, which were identified via de novo motif-discovery analyses. **(D)** Venn diagrams show the overlap between genes bound by OsLEC1 and differentially expressed genes in *Oslec1-1* embryos. Group A indicates 604 genes detected using ChIP-seq and RNA-seq at EE and LE stages; group B indicates 1,226 genes detected using ChIP-seq and RNA-seq at the EE stage; group C indicates 415 genes detected using ChIP-seq and RNA-seq at the LE stage. **(E)** Genome browser view of the chromosomal region showing enrichment of genomic regions bound by OsLEC1 and genes regulated by OsLEC1. The name of the OsLEC1-binding genes are written within the cycle of chromosomes, and genes up-regulated or down-regulated by OsLEC1 are represented by red or blue lines respectively, outside the cycle of chromosomes.

To determine which processes are directly regulated by OsLEC1, we performed chromatin immunoprecipitation followed by high-throughput sequencing (ChIP-seq) using the embryonic callus of *35S:3xFLAG-OsLEC1 2#* and wild type plants. We could not harvest enough young embryos from the *35S:3XFLAG-OsLEC1* transgenic plants to perform ChIP-seq experiment. According to the expression data of *OsLEC1* by GUS staining, embryonic callus seems to be the best alternative tissue besides of the embryo. Expression level of tagged protein and callus formation phenotype of *35S:3xFLAG-OsLEC1* lines are shown in Supplementary Figure 11. The peaks of *35S:3xFLAG-OsLEC1* were distributed mainly at the 5'-UTR sites, in a region within 2 kb upstream of the genes, while the peaks of the wild type did not show a significantly enriched sites and the normalized signal was significantly weaker than that of the *35S:3xFLAG-OsLEC1* (Supplementary Figure 12). In the *35S:3xFLAG-OsLEC1* group, 60.25% of the 27452 peaks were localized in the promoter region, including 51.96% within 1 kb of and 8.29% between 1 and 2 kb of the promoter (Figure 6A). The top enriched KEGG pathways (*p* < 0.05) for these peak targets include amino sugar and nucleotide sugar metabolism (ko00520), butanoate metabolism (ko00650), N-glycan biosynthesis (ko00510), carotenoid biosynthesis (ko00906), and ribosome (ko03010) (Figure 6B, Supplementary Table 12).

DNA sequence motifs that were enriched in LEC1-binding genomic regions of 1 kb upstream of target genes were identified via *de novo* motif-discovery analyses (Figure 6C). These motifs most closely corresponded with several cis-regulatory elements including the G-box (CACGTG) or ABRE-like (C/G/T) ACGTG(G/T) (A/C), which were significantly overrepresented in OsLEC1 target genes with 983 binding sites. We did not detect a CCAAT DNA motif. To determine the genes directly regulated by OsLEC1 in rice seed development, we analyzed the overlapping OsLEC1-binding genes via ChIP-seq, using the DEGs detected in *Oslec1* embryos at EE and LE stages. There were 14,447 unique target genes binding with OsLEC1, as well as 4,154 and 2,861 DEGs at EE and LE stage embryos of *Oslec1* mutants, respectively. Venn diagram showed that OsLEC1 bound to 604 DEGs at EE and LE stages (Figure 6D), which were mainly involved in photosynthesis, chlorophyll biosynthesis, flowering, dehydration resistance, cell wall, heat stress, and hormonal pathways (GA, auxin, ethylene) (Supplementary Tables 13, 14). There were more target genes bound by OsLEC1 specifically at the EE stage (1226) than those at the LE stage (415) (Figure 6D), suggesting that OsLEC1 might control more gene expression pathways in the early stage of seed development. At the EE stage, OsLEC1-binding genes were involved in the ABA pathway, dehydration resistance, lipid metabolic processes, heat stress, cell wall, and other hormonal pathways (auxin, cytokinin, ethylene). At the LE stage, OsLEC1-binding genes were involved in response to stress, seed development, Jasmonate acid (JA), auxin-related and other transcriptional factors (Supplementary Tables 13, 14).

A CIRCOS plot was constructed to identify and analyse similarities and differences between the OsLEC1 target genes according to ChIP-seq results with DEGs at EE and LE stages at the genome scale (Figure 6E). The gene density and number were larger at the EE stage compared with those at the LE stage. According to the annotation and the chromosomal distribution, target genes of OsLEC1 were mapped for all 12 rice chromosomes with similar densities (Figure 6E).

### OsLEC1 Bound to DEGs in Photosynthesis and Seed Maturation Processes

**Figure 7:**
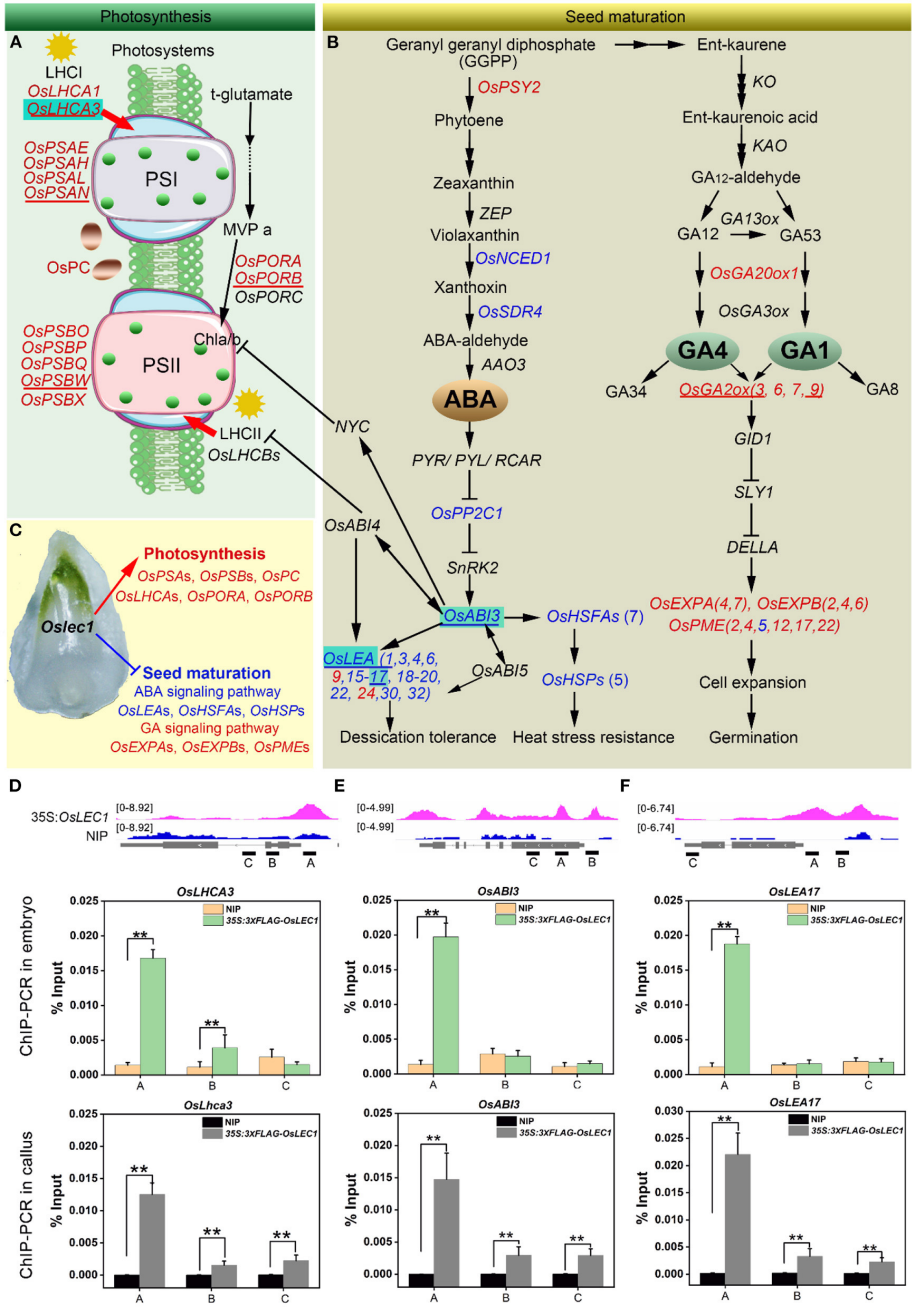
A proposed model of the mutated *Oslec1* action. **(A)** Photosynthesis-related genes were up-regulated in the *Oslec1* mutant, including genes encoding key enzymes PSI, PSII, LHCI, LHCII and OsPC, etc. **(B)** *Oslec1* mutation regulated genes involved in seed maturation, including genes in ABA/GA biosynthesis and signaling pathways. **(C)** *Oslec1* mutation results in the activation of photosynthesis and the inhibition of seed maturation. Genes underlined indicate that they are verified by qRT-PCR. Genes in red indicate that they are upregulated in *Oslec1* embryos, and directly bound by OsLEC1; genes in blue indicate that they are downregulated in *Oslec1* embryos and directly bound by OsLEC1. **(D–F)** IGV screenshot of peak sites on genome sequences of OsLEC1-binding genes and ChIP-qPCR analysis of the binding genes in embryos and callus of *35S:3* × *FLAG-OsLEC1 2#* and the wild type. These three genes are all marked with cyan background in the model above. The asterisks show significant differences (*t*-test; **P < 0.01) between 35S:3 × FLAG-OsLEC1 and the wild type.

According to the results above, we illustrated an OsLEC1-binding/regulating gene network using a combination of RNA-seq and ChIP-seq data. DEGs involved in photosystem I (*OsPSA*s), photosystem II (*OsPSB*s), light-harvesting complex (*OsLHCA*s), electron carrier (*OsPC*), and two genes encoding key enzymes in chlorophyll biosynthesis (*OsPORA* and *OsPORB*), were identified as OsLEC1-binding genes. These genes were upregulated (at least four folds compared with wild type) in *Oslec1* mutant embryos (Figure 7A), possibly contributing to embryo greening (Figure 7C).

In the ABA pathway, we identified *OsNCED1, OsSDR4, OsPP2C1*, and *OsABI3* as OsLEC1-binding genes, which were significantly downregulated in *Oslec1* embryos (Figure 7B). In the downstream of *OsABI3*, 13 *OsLEA*s and 12 *OsHSF*s/*OsHSP90.1* were identified as OsLEC1-binding genes and were significantly downregulated in *Oslec1* embryos (Figure 7B). We also found that *OsGA20ox1* and *OsGA2ox (3, 6, 7, 9)*, which are involved in GA biosynthesis and deactivation, were identified as OsLEC1-binding genes and significantly upregulated in the EE- and LE-stage *Oslec1* embryos (Figure 7B). Cell expansion/cell wall-related genes *OsEXPAs, OsEXPBs*, and *OsPMEs*, downstream of the GA signaling pathway, were also identified as OsLEC1-binding and upregulated genes (Figure 7B). These results suggested that *Oslec1* mutation inhibited the seed maturation processes via inhibiting ABA signaling pathway and promoting GA signaling pathway (Figure 7C).

Integrative genomics viewer (IGV) was used for the straightforward visualization of OsLEC1-binding sites on nine target genes (Figures 7A,B). OsLEC1-binding sites of *OsPSAN, OsPSBW, OsLHCA3, OsPORB, OsLEA1*, and *OsLEA17* were found on the promoter regions, and those of *OsGA2ox3* and *OsABI3* were on the promoters and coding regions, while the binding site of *OsGA2ox9* was mainly on the coding region (Figures 7D,F, Supplementary Figure 13). Among them, *OsLHCA3, OsABI3*, and *OsLEA17* were verified by CHIP-qPCR in both embryos and callus of *35S:3xFLAG-OsLEC1*. The CHIP-qPCR results were consistent with IGV screenshot from the ChIP-seq (Figures 7D–F).

## Discussion

**Figure 8:**
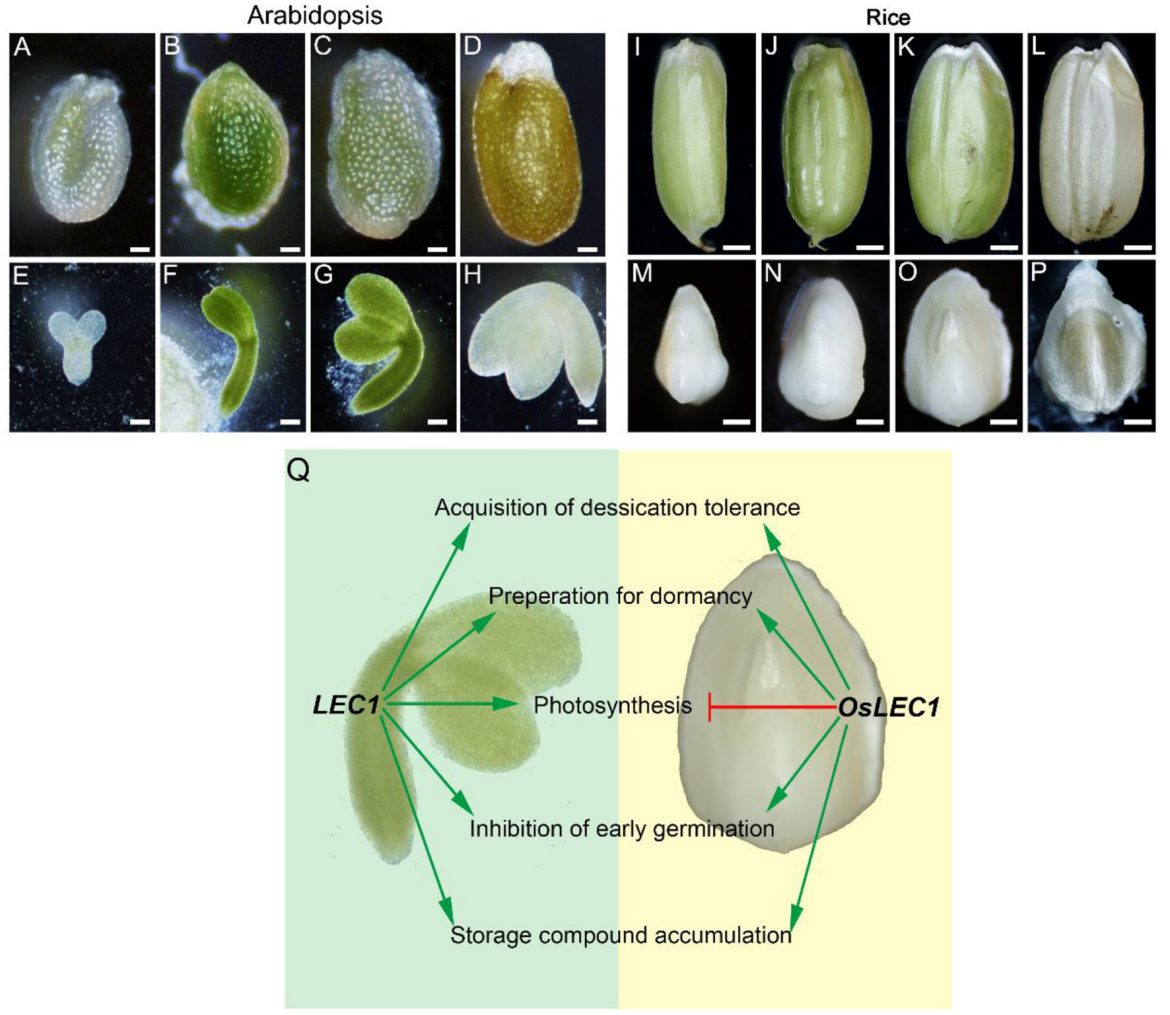
Comparisons of LEC1 functions during seed development in rice and Arabidopsis. **(A–D)** Complete Arabidopsis seeds in different developmental stages. Scale bars = 200 μm. **(E–H)** Arabidopsis embryos in different developmental stages. Scale bars = 50 μm. **(I–L)** Complete rice seeds in different developmental stages. Scale bars = 500 μm. **(M–P)** Rice embryos in different developmental stages. Scale bars = 50 μm. **(Q)** Comparison of LEC1 functions during seed development in rice and Arabidopsis. Arrowed green lines represent promotional effects, and a flat red line show inhibitory effects.

LEC1 is an essential regulator of seed maturation (Meinke et al., 1994; West et al., 1994; Lotan et al., 1998; To et al., 2006; Braybrook and Harada, 2008; Pelletier et al., 2017; Lepiniec et al., 2018; Jo et al., 2019). The studies on LEC1 function are summarized in Supplementary Figure 14. In the present study, we described the resulting seed development phenotypes of the rice *Oslec1* mutant and illustrated an OsLEC1-binding/regulating gene network using a combination of RNA-seq and ChIP-seq data. We found that *Oslec1* mutation did not cause embryo lethality but caused disrupted desiccation tolerance, which caused death due to drying after seed harvesting (Figure 1). Chlorophyll accumulation in *Oslec1* embryos was triggered at a very early stage of seed development (Figure 2). OsLEC1 could directly bind and regulate many genes involved in ABA/GA biosynthesis and signaling pathway, contributing to dormancy (Figure 7). It is widely recognized that phytohormones ABA and GA are the primary hormones regulating seed dormancy and germination, respectively (Finkelstein et al., 2002, 2008; Graeber et al., 2012; Lee et al., 2015). Levels of major storage molecules were dramatically reduced in *Oslec1* mutant embryos (Figure 2). Notably, the shoot apices of *Oslec1* embryos were activated and possessed leaf primordia, whereas wild type embryonic shoot apices were inactive and did not initiate leaf development (Figure 2). Similar phenotypes were reported in Arabidopsis (Meinke, 1992; Meinke et al., 1994; West et al., 1994). A recent study showed that OsNF-YB7 (OsLEC1) complements the developmental defects of *lec1-1* in Arabidopsis when driven by the *LEC1* native promoter (Niu et al., 2021). Therefore, OsLEC1 has a conservative function in seed maturation among dicots and monocots, which is required for the acquisition of desiccation tolerance and seed dormancy, storage compound accumulation, and inhibition of early germination (Figure 8Q).

ABA and GA are major players in seed dormancy and germination regulation (Cutler et al., 2010; Shu et al., 2016). Some clues indicate the interaction of LEC1 and ABA/GA pathways. LEC1-induced embryonic differentiation in vegetative seedlings is strictly dependent on an elevated level of ABA (Junker et al., 2012). LEC1/L1L-(NF-YC2) activation depends on ABA-response elements (ABRE) present in the promoter of Cruciferin C (CRC), which encodes a seed storage protein (Yamamoto et al., 2009), and the ABRE-like motif (C/G/T)ACGTG(G/T)(A/C) was significantly overrepresented in LEC1 target genes (Pelletier et al., 2017). In addition, LEC1 interacts with DELLA proteins, which are GA signaling repressors. GA triggers the degradation of DELLAs to reverse LEC1 repression, thus promoting auxin accumulation to facilitate embryonic development (Hu et al., 2018). Furthermore, LEC1 interacts with different combinations of AREB3, bZIP67, and ABI3 to control diverse biological pathways during soybean seed development (Jo et al., 2020). LUC assay results showed that *NCED1, AREB3, GA3ox1*, and *GA20ox2* are bound and directly regulated by LEC1 (Jo et al., 2020). We showed that OsLEC1 bound to and positively regulated *OsNCED1, OsSDR4, OsPP2C*s, and *OsABI3* in ABA biosynthesis and signaling but negatively regulated *GA2ox3* and *GA2ox9* in the GA pathway (Figure 7B), consistent with previous studies. Taken together, OsLEC1 plays a conservative role in controlling seed maturation through the regulation of ABA and GA pathways.

Owing to the non-photosynthetic nature of the rice seed, the rice embryo remains white during seed development and maturation (Figures 8I–P), in contrast to the Arabidopsis seed which undergoes embryo greening (Figures 8A–H). Notably, *Oslec1* embryos turned green at 7 DAP (Figure 2), showing that photosynthesis had been activated in the very early stages of seed development. Compared with wild type plants, a series of photosynthesis-related genes were significantly upregulated in *Oslec1* embryos (Figures 5A,B), and some of them were also OsLEC1-binding targets (Figure 6E), indicating a suppressive effect of OsLEC1 on photosynthesis (Figure 8Q). The results revealed that OsLEC1 is a key inhibitor of photosynthesis-related genes in rice embryo development.

In Arabidopsis, LEC1 interacts with PHYTOCHROME INTERACTING FACTORs (PIF4) to coactivate genes involved in dark-induced hypocotyl elongation (Huang et al., 2015) and regulate *LHCB* genes through interaction with pirin, a protein that enhances TF binding in mammals (Warpeha et al., 2007). However, the role of LEC1 on photosynthesis was largely unknown. It was reported that maturing embryos of the *lec1* mutant display a paler green than wild type embryos, and most DEGs involved in photosynthesis and chloroplast biogenesis are downregulated in Arabidopsis *lec1* mutant (Pelletier et al., 2017). LUC assay showed that *PSBW* and *PSBP-1*, involved in photosynthesis system II, are activated by LEC1 in soybean embryo cotyledon cells, and LEC1 may interact with different TFs to activate distinct gene sets (Jo et al., 2020). Thus, LEC1 could promote but is not essential for photosynthesis and chloroplast biogenesis in Arabidopsis and soybean seed development (Pelletier et al., 2017; Jo et al., 2020). In contrast, our results show that OsLEC1 blocks photosynthesis in rice seed maturation. An explanation of the differences in the effect of LEC1 on photosynthesis in seed maturation between rice and Arabidopsis possibly lies in the different seed developing processes. Photosynthesis is activated sequentially during Arabidopsis embryo development (Allorent et al., 2013; Hu et al., 2018). Rice seed is non-photosynthetic, and has an endosperm for nutrient supply in the early stage of plant growth, and photosynthesis is completely absent in the embryo during seed development. Thus, the activation of photosynthesis in *Oslec1* mutants was easily observed in rice seed maturation in the present study. *OsLEC1*, a monocot homolog of *LEC1*, blocks photosynthesis in rice embryos. Evidence showed that *OsNF-YB7* (*OsLEC1*) expression could complement the developmental defects of Arabidopsis *lec1-1*, such as the morphology of cotyledons and the desiccation tolerance, but the cotyledons were green, suggesting that *OsLEC1* did not block photosynthesis in Arabidopsis embryos (Niu et al., 2021). This implies that, other genes, which controlling photosynthesis together with *LEC1*, also control Arabidopsis embryo maturation and greening. It is noteworthy that the mutations of OsLEC1 are present downstream of the DNA-binding domain, which could still be expected to generate a truncated protein (about 157 aa) including the DNA-binding domain (Supplementary Figure 1). Thus, we could not exclude the possibility that the mutations of OsLEC1 may have some residual or even additional functions. Further experiments are necessary to confirm the function of OsLEC1, for example, to construct a transgenic line with the *OsLEC1* driven by its endogenous promoter in the *Oslec1* mutant, or reconstruct the knockout lines of *OsLEC1* (by designing the gRNA in the upstream of the DNA binding domain). In addition, differences in the conserved region of the LEC1 protein sequence also suggested possible functional differences between LEC1 in dicots and monocots (Supplementary Figure 1). Therefore, we speculate that the role of OsLEC1 in rice is not identical to that of LEC1 Arabidopsis in photosynthesis (Figure 8Q).

In our ChIP-seq results, the G-box motif (CACGTG), was significantly overrepresented in all OsLEC1-target gene sets identified (Figure 6C), similar with the results in Arabidopsis (Pelletier et al., 2017). However, CCAAT motif, a known binding site of the LEC1 NF-Y complex, was not found in ChIP-seq binding results from callus. It was reported that the CCAAT-binding sequence motif bound by LEC1 was significantly overrepresented only in the Arabidopsis BCOT-stage seeds, but not in early and late-stage seeds (Pelletier et al., 2017). Binding sequences of LEC1 vary in different tissues and developmental stages. Thus, we verified that *OsLHCA3, OsABI3*, and *OsLEA17* bound by OsLEC1 in embryos, consistent with data from callus (Figures 7D–F). Therefore, the sequence motifs bound by LEC1 in embryos were, at least in part, similar to those in the callus. It is necessary to further study the role of OsLEC1-binding targets in rice embryos during the seed development.

In conclusion, our findings suggest that OsLEC1 is an inhibitor of embryo greening during seed development and a central regulator of seed maturation. Revealing the underlying mechanism of how OsLEC1 regulates seed maturation would help to generate strategies to enhance grain dormancy levels and prevent PHS (pre-harvest sprouting) of crop species. Breakthroughs regarding OsLEC1 regulatory mechanisms on photosynthesis would expand our understanding of the molecular network underlying photosynthesis, thus contributing to improving photosynthesis in agriculture.

## Methods

### Plant Materials and Growth Conditions

Wild type “Nipponbare” rice (*Oryza sativa* L. ssp. *japonica*) and Columbia-0 (Col-0), as the wild type Arabidopsis, were used. *Oslec1, pOsLEC1:GUS, 35S:OsLEC1* mutants were all generated in the “Nipponbare” background. Plants were grown in a greenhouse under a 16 h light, 30°C/8 h dark, 24°C cycle.

For callus induction, sterile seeds were placed in callus induction medium (N6 basal medium supplemented with 10 μM 2,4-D, pH 5.8) and incubated in the growth chamber under a 16 h light, 28°C/8 h dark, 24°C cycle (Guo et al., 2018).

For the germination assay, rice seeds (dry or wet) were unshelled and soaked in distilled water at 30°C in the dark until they germinated. Protruded seeds were considered germinated seeds. Then, uniformly germinated seeds were placed in Petri dishes (12 cm) covered by filter paper soaked with distilled water and grown at 28°C and 90% relative humidity under 16 h light/8 h dark conditions.

### Plasmid Construction and Rice Transformation

*Oslec1* transgenic plants were generated using CRISPR-Cas9 technology, according to a previously described method (Xie et al., 2015). We designed two single gRNAs that specifically targeted the protein-coding regions of *OsLEC1*. The two gRNAs were assembled into a single vector using the polycistronic-tRNA-gRNA (PTG) strategy. Two gRNAs 5′-CTCTGGGCCATGAGCCGCCT-3′ and 5′-CAGACCGTCAACTTCGAGCA-3′ were assembled into a single vector *pRGEB32* using the polycistronic-tRNA-gRNA (PTG) strategy to construct *OsLEC1-PRGEB32*.

To generate the *pOsLEC1:GUS*, a 2841 bp genomic sequence upstream the ATG start codon of *OsLEC1* (*LOC_Os02g49370*) was cloned from the rice genome, and inserted into the *pENTR/D-TOPO* vector (Invitrogen, Carlsbad, CA, USA), and subsequently into the destination vector *pHGWFS7*, which contained a β-glucuronidase (GUS) gene fusion created via LR Clonase (Thermo Fisher Scientific, Waltham, MA, USA) reactions.

For the ChIP assay, 35S:3^*^FLAG-OsLEC1 was constructed first by fusing a sequence encoding the 3^*^FLAG peptide sequence with the *OsLEC1* cDNA, and the fusion was then inserted into pCAMBIA 1300 using infusion cloning according to the manufacturer's instructions.

The vectors *OsLEC1-PRGEB32, pOsLEC1:GUS*, and 35S:3^*^FLAG-OsLEC1 were introduced into rice callus using the *Agrobacterium tumefaciens*-mediated co-cultivation approach through the EHA105 strain. Homozygous T3 seeds were used for further experiments.

For subcellular co-localization of proteins, the coding sequence, not including the stop codon of OsLEC1, was cloned into the pUGW5 vector to generate the *35S:OsLEC1-GFP* via LR Clonase reactions according to the manufacturer's instructions. The *35S:OsIAA1-mcherry* vector has been described previously (Guo et al., 2021). The primers used are listed in Supplementary Table 15.

### SEM, TEM, and Light Microscopy

For SEM analysis, 5-DAP and 25-DAP embryos of NIP and *Oslec1* were fixed overnight at 4°C in 2.5% glutaraldehyde in phosphate buffer (0.1 M, pH 7.0), washed three times in the phosphate buffer (0.1 M, pH 7.0) for 15 min at each step, then, postfixed with 1% OsO_4_ in phosphate buffer for 2 h and washed three times in phosphate buffer. The samples were dehydrated through an ethanol gradient (30, 50, 70, 80, 90, 95, and 100% ethanol, 15 min each), then transferred to absolute ethanol, and dehydrated in Hitachi Model HCP-2 critical point dryer (Hitachi, Tokyo, Japan). The dehydrated sample was coated with gold-palladium in Hitachi Model E-1010 ion sputter for 4–5 min and observed using the Hitachi Model SU-8010 SEM (Hitachi, Tokyo, Japan).

For TEM, the samples were fixed in 2.5% glutaraldehyde and postfixed with 1% OsO4, then, dehydrated through an ethanol gradient, similar to the pre-treatment of SEM samples. After dehydration, samples were immersed in absolute acetone for 20 min, then, into 1:1 and 1:3 mixtures of absolute acetone and the final Spurr resin mixture for 1–3 h, respectively. Then, to the final Spurr resin mixture overnight.

Samples were placed in an Eppendorf containing Spurr resin and heated to 70^o^C for more than 9 h, then, sectioned in a LEICA EM UC7 ultratome. Sections were stained with uranyl acetate and alkaline lead citrate for 5 and 10 min, respectively, and observed using the Hitachi Model H-7650 TEM.

For light microscopy, the embedded samples were sectioned at 2–5 μm thickness using a rotary microtome (Leica, Wetzlar, Germany) and stained with basic fuchsin and observed using a Nikon C-C phase turret condenser (Nikon, Tokyo, Japan).

### Histochemical Analysis of GUS Activity and TTC Staining

For GUS staining, *pOsLEC1:GUS* embryos were incubated in the X-Gluc solution overnight at 37°C, as described previously (Guo et al., 2016) and then fixed in 2.5% glutaraldehyde overnight to prepare them for imaging or semi-thin sectioning.

For TTC staining, the seeds were split longitudinally and soaked in 1% (w/v) TTC solution at 37°C for 3 h, as described previously (Zhang et al., 2020).

### Subcellular Localization of OsLEC1

Rice protoplasts were prepared and transformed as previously described (Bai et al., 2014). Approximately 8 μg of the expression vector *35S:OsLEC1-GFP* was transferred into rice protoplasts, then, the protoplasts were cultured at room temperature in the dark for 16 h. Fluorescence signals were detected and photographed using an LSM710 NLO confocal laser scanning microscope (Zeiss, Mannheim, Germany). Excitation/emission wavelengths were 488 nm for GFP and 561/575–630 nm for mCherry. Fluorescence signals were analyzed using the Zen2009 (Carl Zeiss) software.

### RNA Extraction and qRT-PCR Analysis

Total RNA was extracted from *Oslec1* and wild type embryos using the TRIZOL reagent (Invitrogen, Carlsbad, CA, USA) and cDNA was synthesized from 1 μg of total RNA using ReverTra Ace^TM^ qPCR RT Master Mix with gDNA Remover (TOYOBO, Osaka, Japan). qRT-PCR analysis was performed on a Mastercycler ep realplex system (Eppendorf, Hamburg, Germany) using LightCycler 480 SYBR Green Master Mix (Roche, Indianapolis, USA). *OsUBQ5* was amplified and used as an internal standard to normalize the expression of tested genes. Experiments were performed in triplicate. The primers of the examined genes are listed in Supplementary Table 15.

### RNA-Seq Analysis

We collected about 30 embryos of *Oslec1* and wild type (7 and 25 DAP) and extracted mRNAs to perform Illumina sequencing. Three biological replicates were conducted. Library construction and deep sequencing were carried out using the Illumina Hiseq 2500 (Biomarker Technologies, Beijing, China). The adapters and low-quality reads were removed using Trimmomatic (version 0.36) (Bolger et al., 2014). Afterwards, the clean reads were mapped to the rice reference genome (Oryza_sativa. IRGSP-1.0.45) using Hisat2 (version 2.1.0) (Kim et al., 2015). Transcripts were assembled and merged using StringTie (version 1.3.4d) (Pertea et al., 2015). DEGs were identified using DESeq2 (version 1.26.0) (Love et al., 2014), with a false discovery rate of <0.05 and an absolute value of log2 (fold change) > 2.

The GO terms and KEGG enrichment analyses were performed using the online platform g:Profiler (https://biit.cs.ut.ee/gprofiler/gost) (Raudvere et al., 2019). The directed acyclic graphs of GO terms were constructed using the online platform AgriGO v2.0 (http://systemsbiology.cau.edu.cn/agriGOv2/) (Tian et al., 2017). The heat-map of the DEGs were drawn using the online platform Omicstudio (https://www.omicstudio.cn/login). Construction of the GO term network was performed using Cytoscape (Shannon et al., 2003). The RNA-seq data have been deposited in the Gene Expression Omnibus (GEO) database, www.ncbi.nlm.nih.gov/geo (Accession No: GSE179838).

### ChIP, ChIP-QPCR, and ChIP-Seq Analysis

ChIP was performed as previously described (Xu et al., 2008). About 3 g of the wild type and 35S:3^*^FLAG-OsLEC1 callus were collected and treated with 1% formaldehyde for protein-DNA cross-linking. After fixation, chromatin was sonicated with Diagenode Bioraptor to generate 200–1,000 bp fragments. Chromatin was immunoprecipitated with anti-DDDDK monoclonal antibody (MBL, Beijing, China). Then, chromatin-antibody complexes were precipitated with anti-IgG paramagnetic beads (GE Healthcare, Uppsala, Sweden). After six washing steps, complexes were eluted and reverse-crosslinked. ChIP-qPCR was performed with three biological replicates, and each replicate was tested with three technical repeats. Three pairs of specific primers were designed for each target gene, based on different genomic sites. The primer sequences for ChIP-qPCR are listed in Supplementary Table 15. The results were normalized to the input control as previously reported (Asp, 2018).

DNA fragments from ChIP experiment were sent to Genergy Biotechnology (Shanghai, China) for ChIP-seq. ChIP-seq libraries were prepared using Ovation Ultralow Library Systems (Nugen) according to the manufacturer's instructions. Sequencing reads of the ChIP-seq were aligned using Bowtie2 (Langmead and Salzberg, 2012) against the Oryza sativa IRGSP-1.0 genome assembly, and only uniquely mapped sequencing reads were retained. MACS2 (Zhang et al., 2008) was used to call peaks compared to the input using q_value_thresholds=0.01. The aligned reads with biological replicates were processed based on the irreproducibility discovery rate (IDR) (Li et al., 2011). Peaks were then annotated using ChIPseeker (Yu et al., 2015). MEME-ChIP (Machanick and Bailey, 2011) suite was used to identify the DNA binding motif. Visualization of peaks on genomic regions was achieved with IGV 2 (Thorvaldsdóttir et al., 2013). OsLEC1-binding peak positions according to the ChIP-seq results and the DEGs at EE and LE stages between the *Oslec1* mutant and the wild type plants were visualized using CIRCOS (Wyatt et al., 2013). The ChIP-seq data have been deposited in the Gene Expression Omnibus (GEO) database, www.ncbi.nlm.nih.gov/geo (Accession No: GSE179596).

### Western Blot Analysis

About 0.1 g callus from *35S:3XFLAG-OsLEC1* seeds cultured on CIM for 30 days were ground in liquid nitrogen and resuspended in the extraction buffer as previously reported (Zhang et al., 2020). Then the protein samples were resolved by 4–20% gradient SDS-PAGE and transferred to a 0.2-μm polyvinylidenefluoride membrane (Millipore). Proteins were detected with a primary anti-FLAG antibody and incubated with a secondary antibody as previously reported (Guo et al., 2021).

### Protein Sequences Analysis

Protein sequences of the *LEC1* homologous genes in dicots and monocots were obtained from the National Center for Biotechnology Information database (https://www.ncbi.nlm.nih.gov/). The phylogenetic tree of the protein sequences was constructed with MEGA (https://www.megasoftware.net/) using the NJ method with the following parameters: Poisson correction, complete deletion, and bootstrap (1,000 replicates, random seed). Multiple sequence alignments of proteins were performed using the ALIGNMENT software (https://www.genome.jp/tools-bin/clustalw).

## Data Availability Statement

The datasets presented in this study can be found in online repositories. The names of the repository/repositories and accession number(s) can be found below: National Center for Biotechnology Information (NCBI) BioProject database under accession number GSE179596.

## Author Contributions

FG, HB, MC, and LX designed the research. PZ and YW performed the data analyses. GL, WL, BB, CZ, DL, ZY, and WZ performed the experiments. FG and HB wrote the manuscript. NH, ZT, MZ, MC, and LX revised the manuscript. All authors contributed to the article and approved the submitted version.

## Funding

This research was supported by the grants from the National Natural Science Foundation of China (Grant Nos. 31971932 and 31771477), Science Foundation of Zhejiang Province (Grant No. LGN21C130006), Sponsed by Research Startup Funding from Hainan Institute of Zhejiang University (NO. 0201-6602-A12202), the State Key Laboratory of Subtropical Silviculture (No. KF2017-09), and China Agriculture Research System (CARS-05-05A).

## Conflict of Interest

The authors declare that the research was conducted in the absence of any commercial or financial relationships that could be construed as a potential conflict of interest.

## Publisher's Note

All claims expressed in this article are solely those of the authors and do not necessarily represent those of their affiliated organizations, or those of the publisher, the editors and the reviewers. Any product that may be evaluated in this article, or claim that may be made by its manufacturer, is not guaranteed or endorsed by the publisher.
