# Supplementary material for: Rice LEAFY COTYLEDON1 Hinders Embryo Greening During the Seed Development
Source: Front Plant Sci. 2022 May 10;13:887980. doi: 10.3389/fpls.2022.887980 (PMC9128838; doi:10.3389/fpls.2022.887980)
Supplement: Supplementary file 2 [file Data_Sheet_1.docx]

Rice LEAFY COTYLEDON1 hinders embryo greening during the seed development

Fu Guo, Peijing Zhang, Yan Wu, Guiwei Lian, Zhengfei Yang, Wu Liu, Buerte B, Chun Zhou, Wenqian Zhang, Dandan Li, Ning Han, Muyuan Zhu, Lin Xu, Ming Chen, Hongwu Bian

The following Supporting Information is available for this article:

**Fig. S1** Phylogenetic relationship and sequence alignment of LEC1 homologous proteins in dicots and monocots and the OsLEC1 protein sequence in the *Oslec1* mutant.

**Fig. S2** Plant architecture and seed phenotype of the *Oslec1* mutant**.**

**Fig. S3** Wild type and *Oslec1* mutant seeds after 24-h imbibition.

**Fig. S4** A close-up of *Oslec1* embryos.

**Fig. S5** Expression pattern of OsLEC1.

**Fig. S6** GUS activity in *OsLEC1pro:GUS* transgenic plants.

**Fig. S7** Spearman correlation between 12 wild type and *Oslec1* mutant samples.

**Fig. S8** GO term analysis of upregulated and downregulated genes in LE-stage *Oslec1* embryos.

**Fig. S9** Differentially-expressed genes in *Oslec1* mutant embryos in two stages involved in CK, BR, ethylene, and auxin pathways.

**Fig. S10** qRT-PCR analysis of transcription levels of OsLEC1-regulated genes.

**Fig. S11** Callus formation phenotype of OsLEC1 overexpressed transgenic lines.

**Fig. S12** Distribution of ChIP-seq reads on gene bodies and upstream/downstream sequences.

**Fig. S13** IGV screenshot of peak sites on genome sequences of OsLEC1-binding genes.

**Fig. S14** A summary of studies that report the functions of OsLEC1.

**Table S1** A list of upregulated genes in EE-stage *Oslec1* embryos.

**Table S2** A list of downregulated genes in EE-stage *Oslec1* embryos.

**Table S3** A list of upregulated genes in LE-stage *Oslec1* embryos.

**Table S4** A list of downregulated genes in LE-stage *Oslec1* embryos.

**Table S5** KEGG pathways in EE and LE stage embryos.

**Table S6** GO term analysis of upregulated genes in EE-stage *Oslec1* embryos.

**Table S7** GO term analysis of downregulated genes in EE-stage *Oslec1* embryos.

**Table S8** GO term analysis of upregulated genes in LE-stage *Oslec1* embryos.

**Table S9** GO term analysis of downregulated genes in LE-stage *Oslec1* embryos.

**Table S10** A list of photosynthesis- and photomorphogenesis-related genes differentially expressed in EE-stage and LE-stage *Oslec1* embryos.

**Table S11** A list of hormones and stress-related genes differentially expressed in EE- and LE-stage *Oslec1* embryos.

**Table S12** KEGG pathway analysis of OsLEC1-binding genes.

**Table S13** Overlapping gene regions according to RNA-seq and ChIP-seq results.**Table S14** All overlapping genes according to RNA-seq and ChIP-seq results.


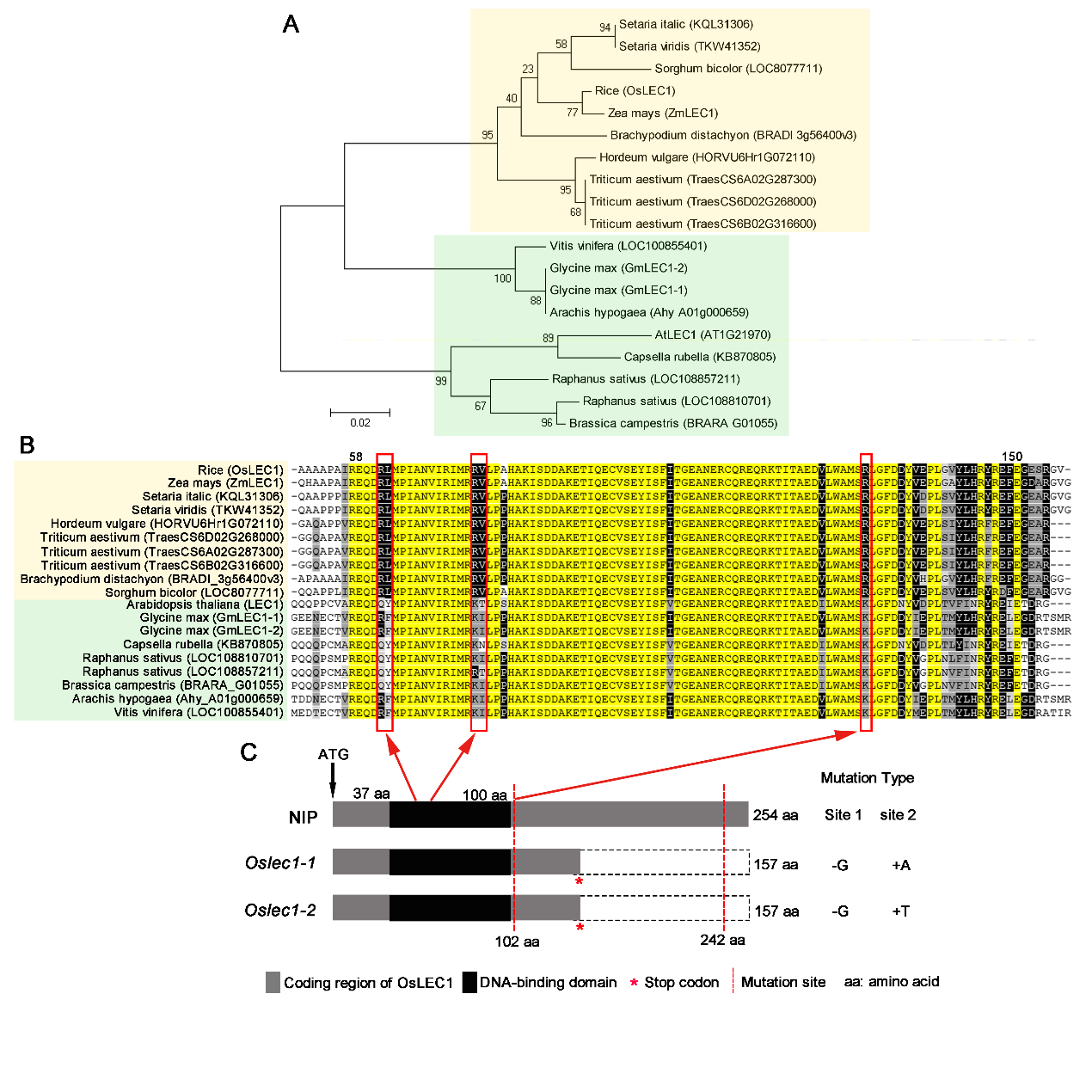
**Fig. S1.** Phylogenetic relationship and sequence alignment of LEC1 homologous proteins in dicots and monocots and the OsLEC1 protein sequence in the *Oslec1* mutant. **(A)** Phylogenetic analysis of LEC1 homologous proteins in dicots and monocots. **(B)** Sequence alignment of LEC1 homologous proteins. LEC1 naturally divided into three clades, indicating it diverged among the different clades. Multiple alignment results showed that LEC1 sequences harbored one conserved domain between amino acid (aa) 31–123 in rice, and the aa identity within the conserved domain reached up to 100%. While there were three sites in the conserved domain, it showed distinct differences between monocots and dicots: Arg 35-Leu 36 (RL); Arg 48-Val 49 (RV); Arg 102(R). The dicots and monocots are shown in light green and light yellow backgrounds, respectively. Identical residues are shown with white letters on a black background; conserved residues are shown with white letters on a grey background; a block of similar and weakly similar residues are shown with black letters on a light grey or white background. Red boxes indicate differential sequences in the conservative region between dicots and monocotyledons. **(C)** OsLEC1 protein sequence in the *Oslec1* mutant. The grey box with 254 aa represents the complete sequence of OsLEC1. The red asterisks indicate the premature translational termination site, the dotted blank boxes indicate the missing aa sequence and the red arrows indicate the differential sequences in the red boxes in B.


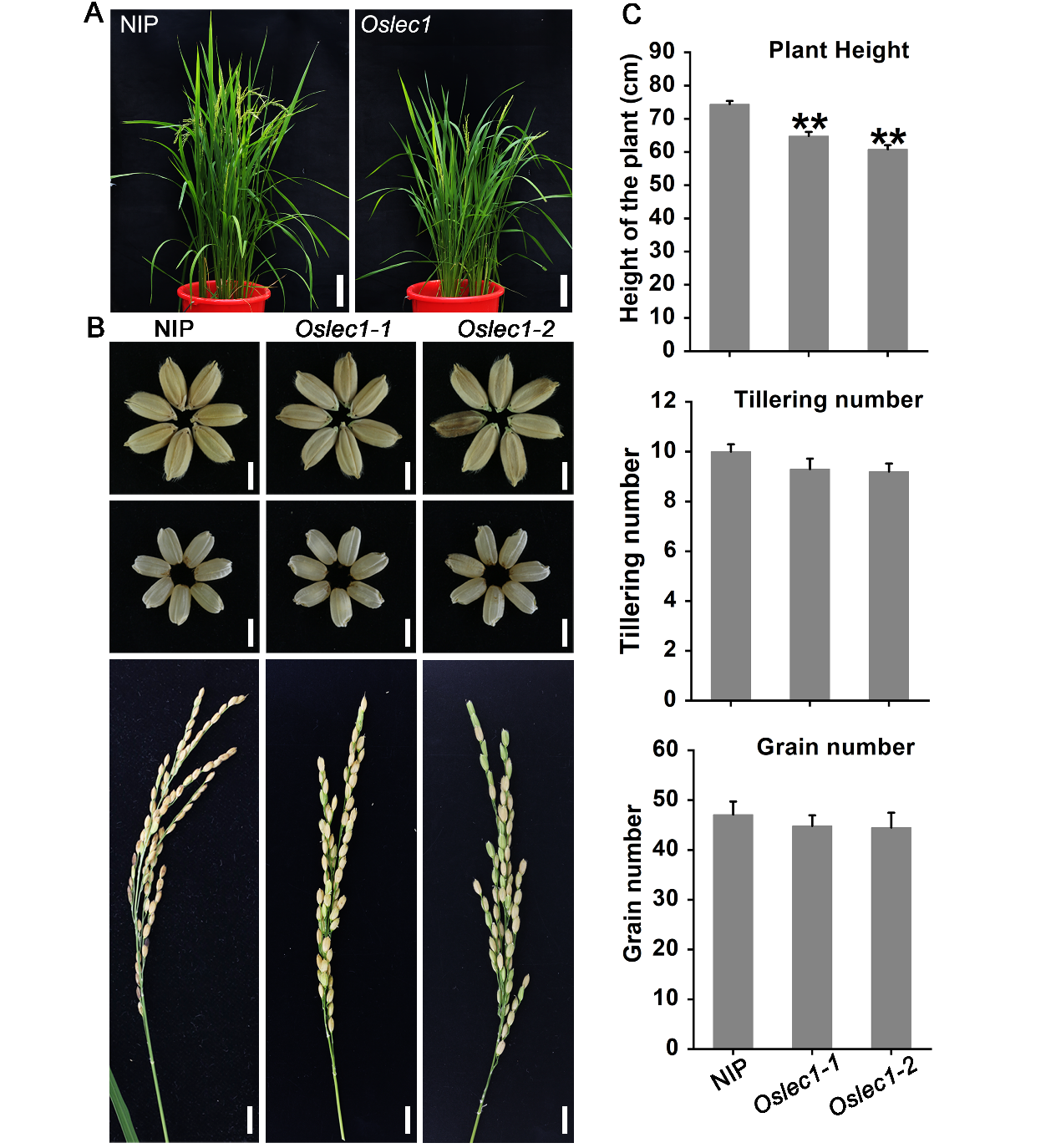


**Fig. S2.** Plant architecture and seed phenotype of the *Oslec1* mutant**. (A)** Architecture of the wild type plant and the *Oslec1* mutant about 3 months after germination. Scale bars=10 cm. **(B)** Mature seeds (Scale bars=5 mm) and panicles (Scale bars=1 cm) of the wild type plant and the *Oslec1* mutant*.* **(C)** Statistics of plant height, tillering number, and grain number/panicle of the wild type plant and the *Oslec1* mutant*.* Error bars indicate the SE of the mean; *P < 0.05; **P < 0.01 (Student’s t-test), n>10.


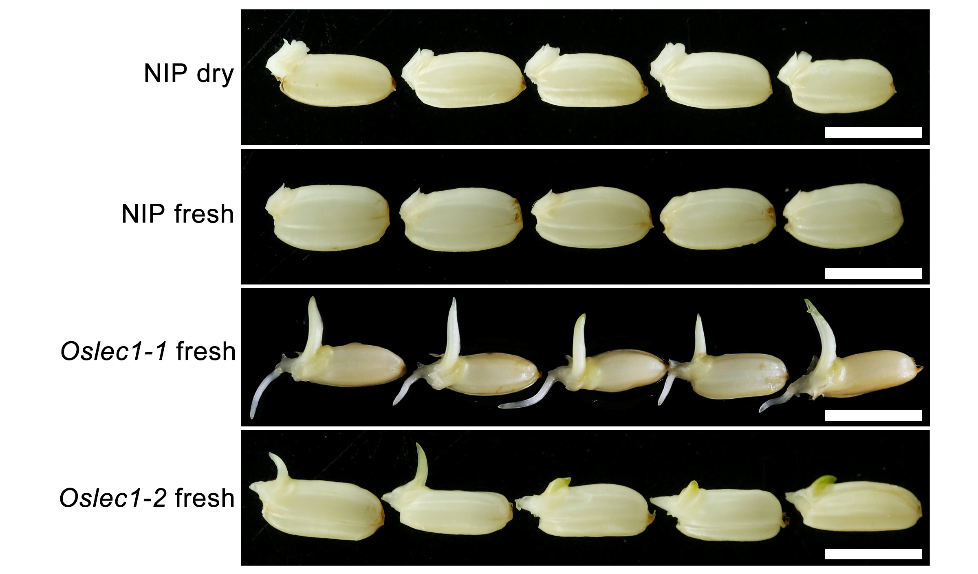


**Fig. S3.** Wild type and *Oslec1* mutant seeds after 24-h imbibition. Coleoptiles have just sprouted from dry and fresh seeds of NIP after 24-h imbibition, while roots have sprouted and the coleoptiles have turned green in fresh seeds of *Oslec1* mutants. Scale bars=5 mm.


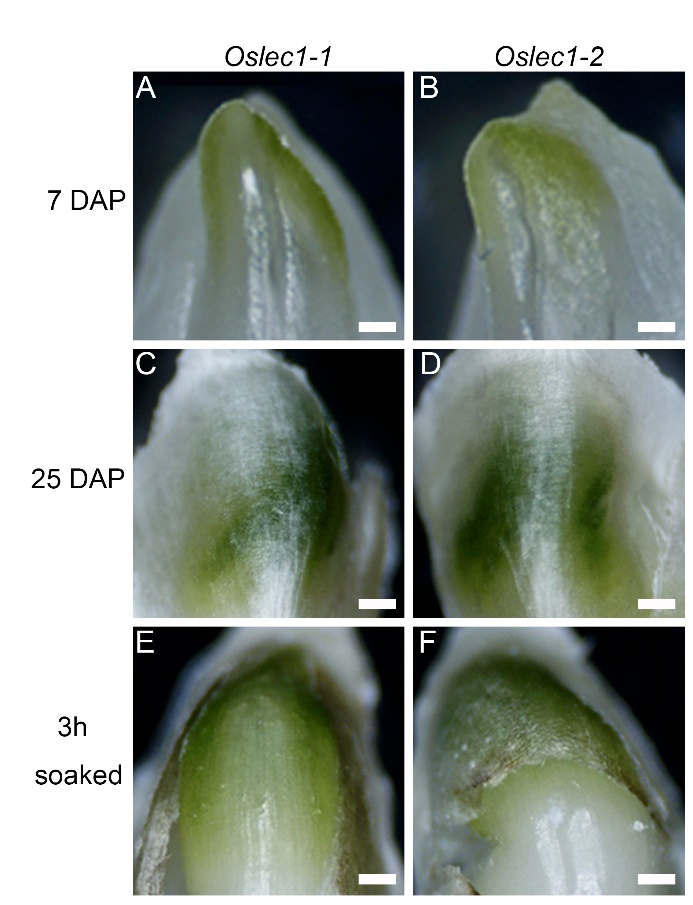


**Fig. S4.** A close-up of *Oslec1* embryos. Close up of *Oslec1* embryos 7-DAP (A-B), 25-DAP (C-D) and embryos of mature seeds soaked for 3 hours in 37℃ (E-F). Bars, 100 µm.


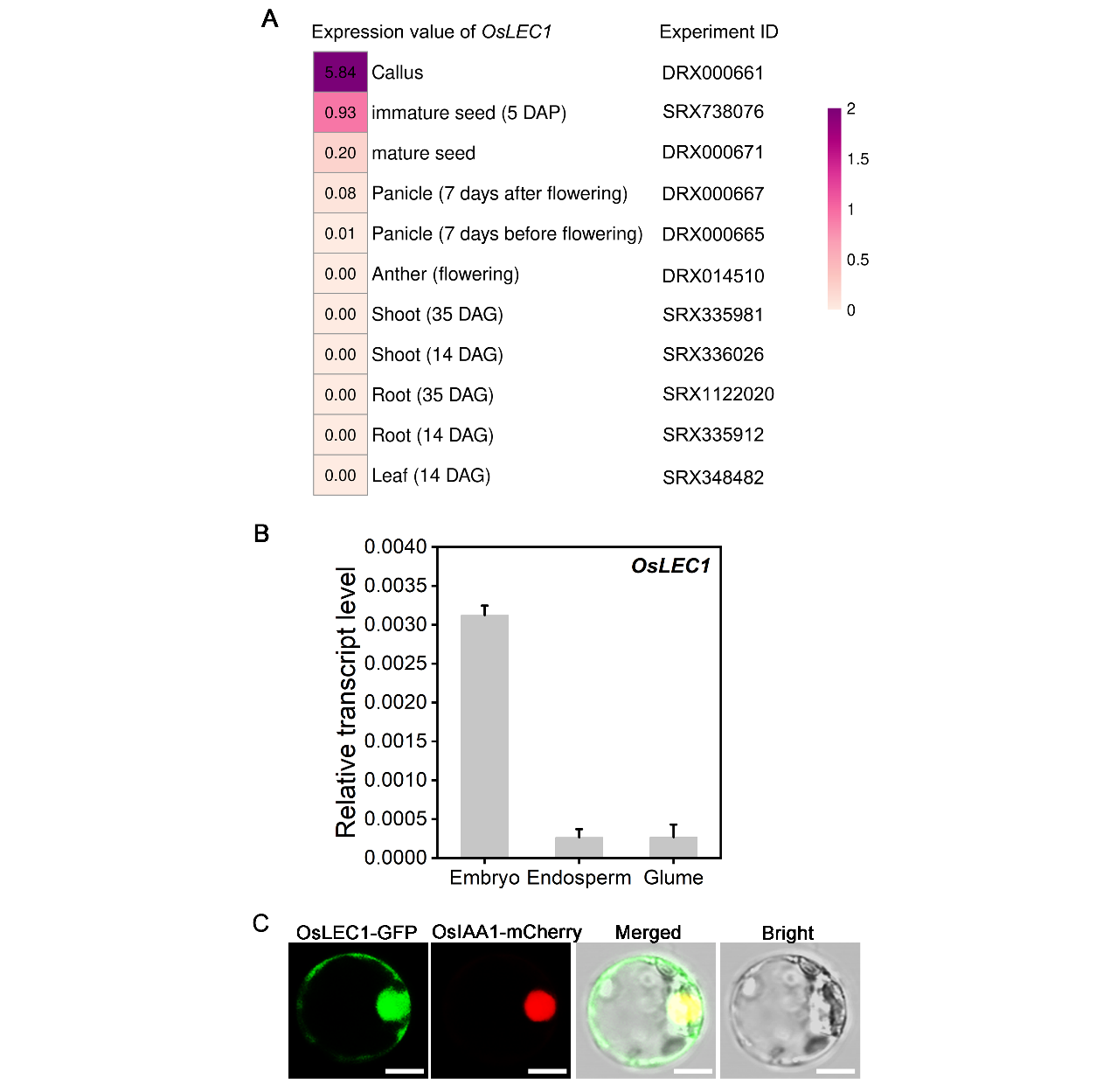


**Fig. S5.** Expression pattern of OsLEC1. **(A)** Expression of *OsLEC1* in different tissues and different stages. The data were obtained from [http://http://expression.ic4r.org/](http://http:/expression.ic4r.org/).**(B)** Transcript levels of *OsLEC1* in different parts of rice seed. Experiments were conducted using three biological replicates, and each replicate was tested with three technical repeats. The results were normalized to the expression of rice *Ubiquitin 5* (*OsUBQ5*). **(C)** Co-localization of OsLEC1-GFP with OsIAA1-mcherry. Both *35S:LEC1-GFP* and *35S: OsIAA1-mcherry* vectors were transformed into rice protoplasts. Fluorescence signals were observed under a confocal microscope. Scale bars=10 µm.


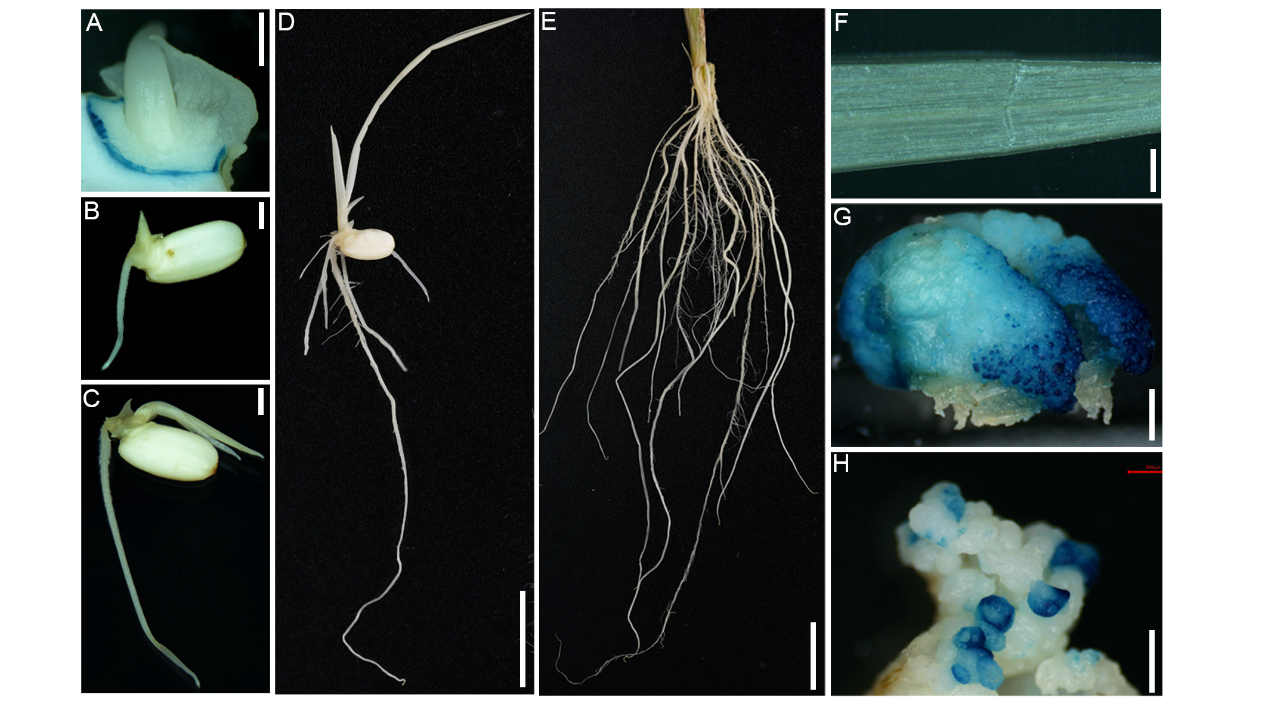


**Fig. S6.** GUS activity in *OsLEC1pro:GUS* transgenic plants. GUS staining results in embryos of germinating seeds (A), 1-DAG (B), 4-DAG (C) and 7-DAG (D) seedlings, roots and leaves of 14-DAG seedlings (E-F), and callus cultured on CIM for 7 and 10 days (G-H). Bars, (A) 500 µm, (B-C) 1 mm, (D-E) 1 cm, (F-H) 500 µm.


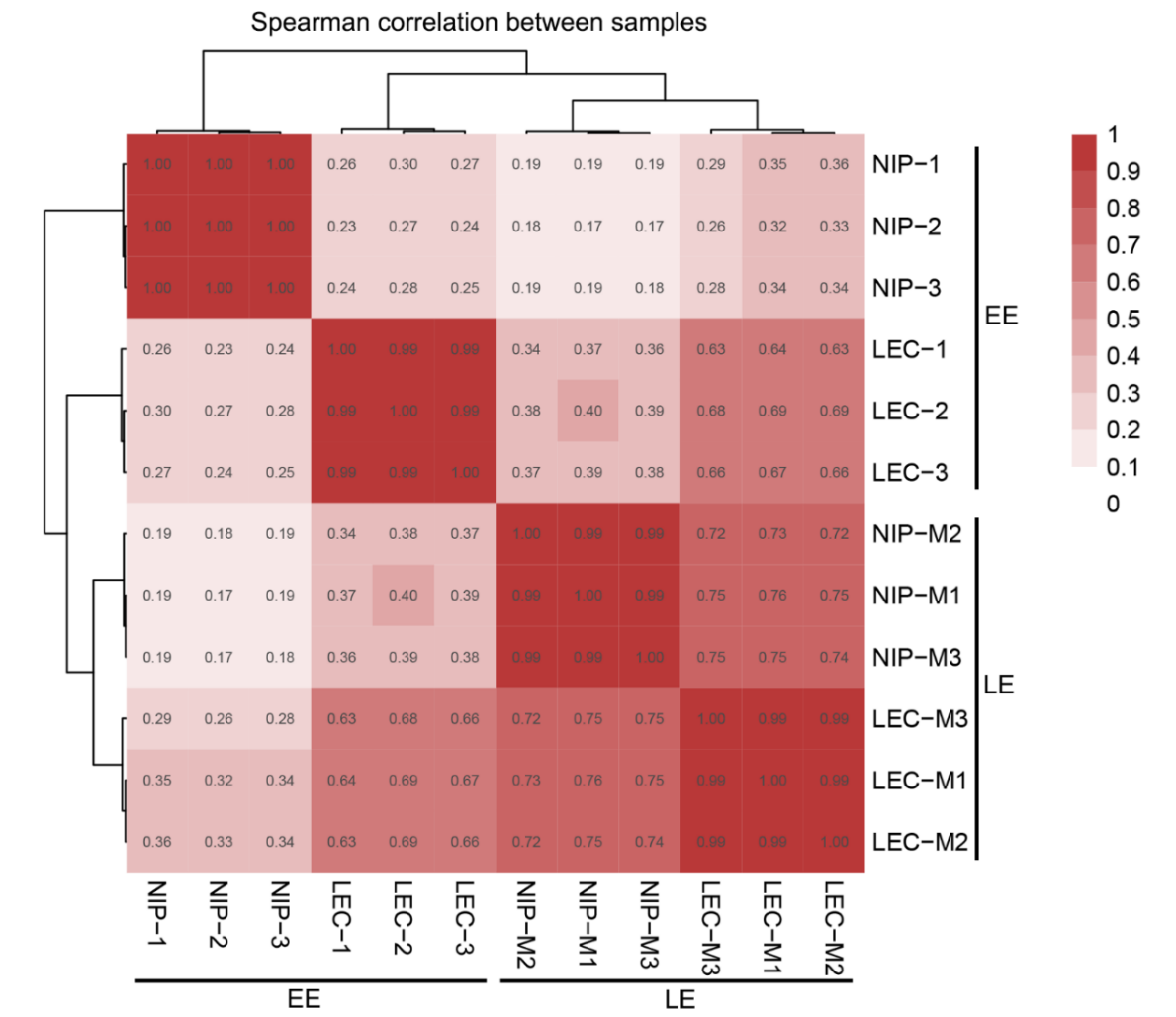


**Fig. S7.** Spearman correlation between 12 wild type and *Oslec1* mutant samples.

EE and LE represents the early and late stage of embryo, respectively. NIP1-3 and LEC1 1-3 are embryos of *Oslec1* mutant and the wild type in EE-stage, NIP-M1, M2, M3 and LEC1-M1, M2, M3 are embryos of *Oslec1* mutant and the wild type in LE-stage.


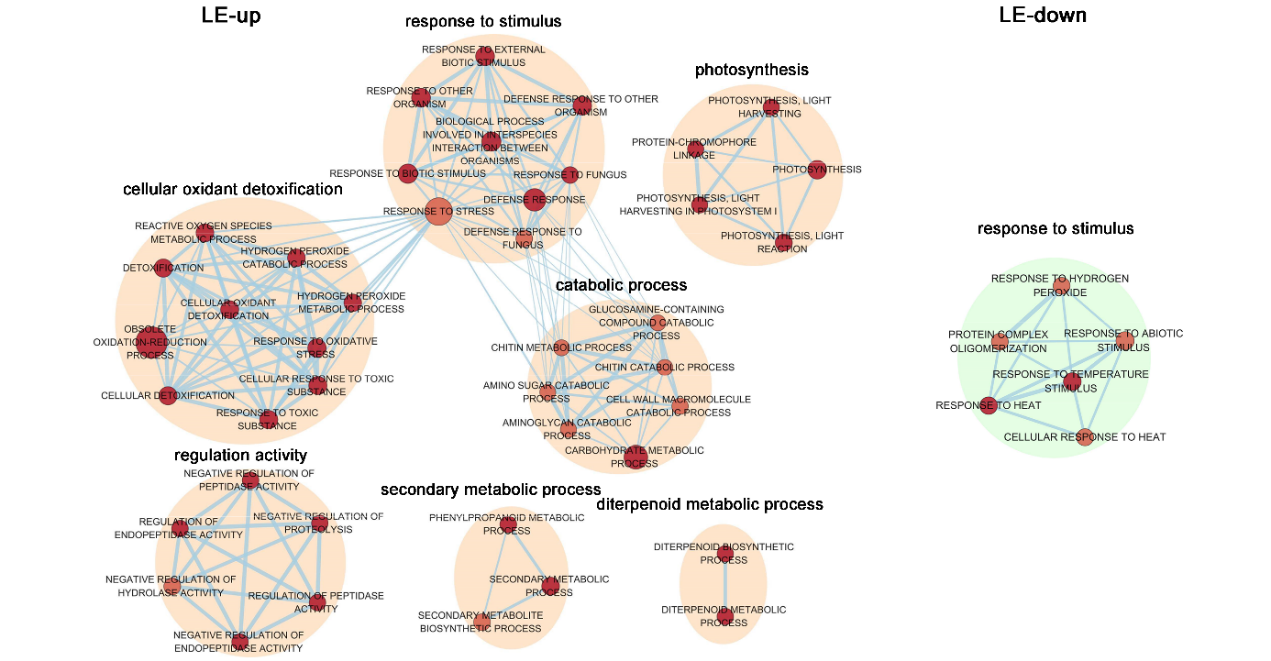


**Fig. S8.** GO term analysis of upregulated and downregulated genes in LE-stage *Oslec1* embryos. GO terms in orange and green background indicate up-regulated and down-regulated biological process, respectively.


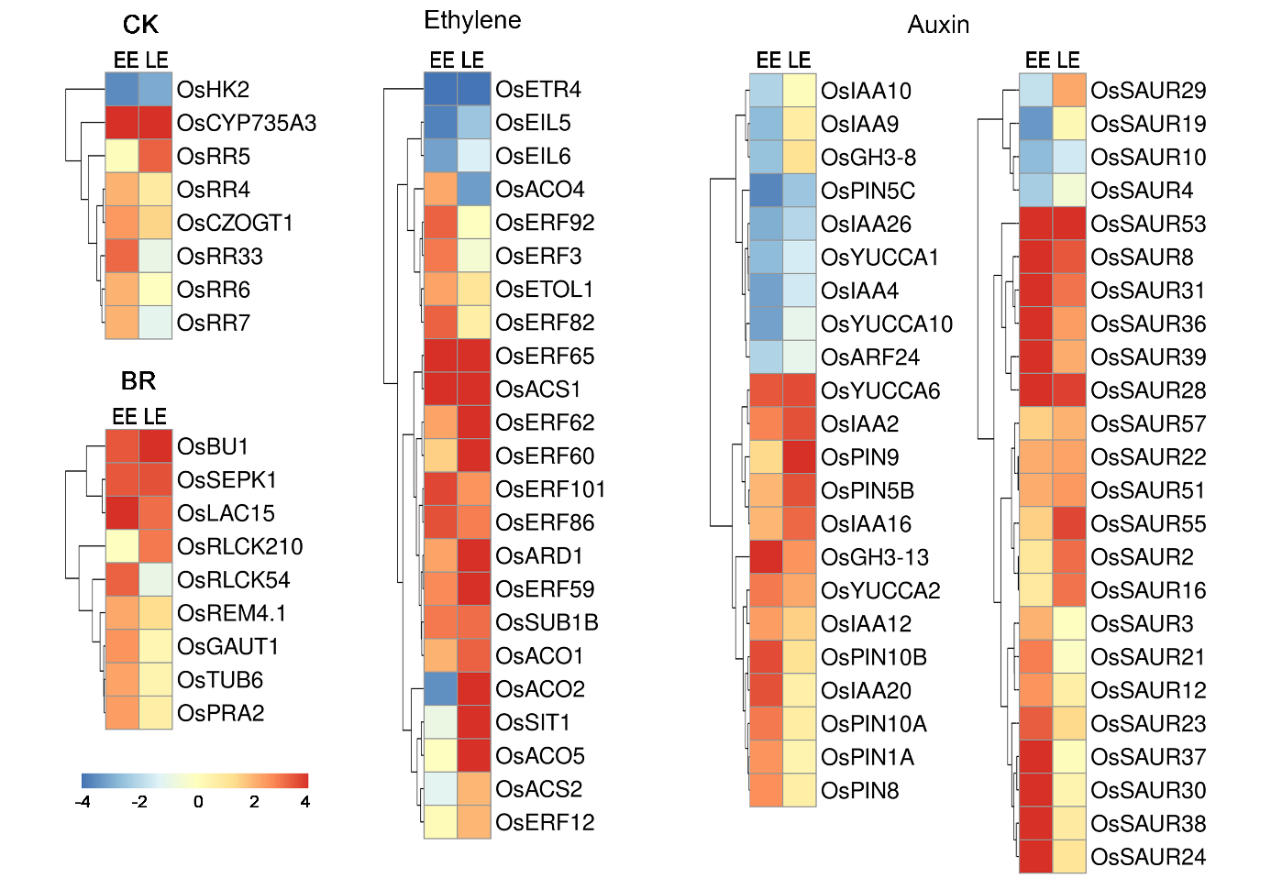


**Fig. S9.** Differentially-expressed genes in *Oslec1* mutant embryos in two stages involved in cytokinin, brassinolide, ethylene, and auxin pathways. CK: cytokinin, BR: brassinolide.


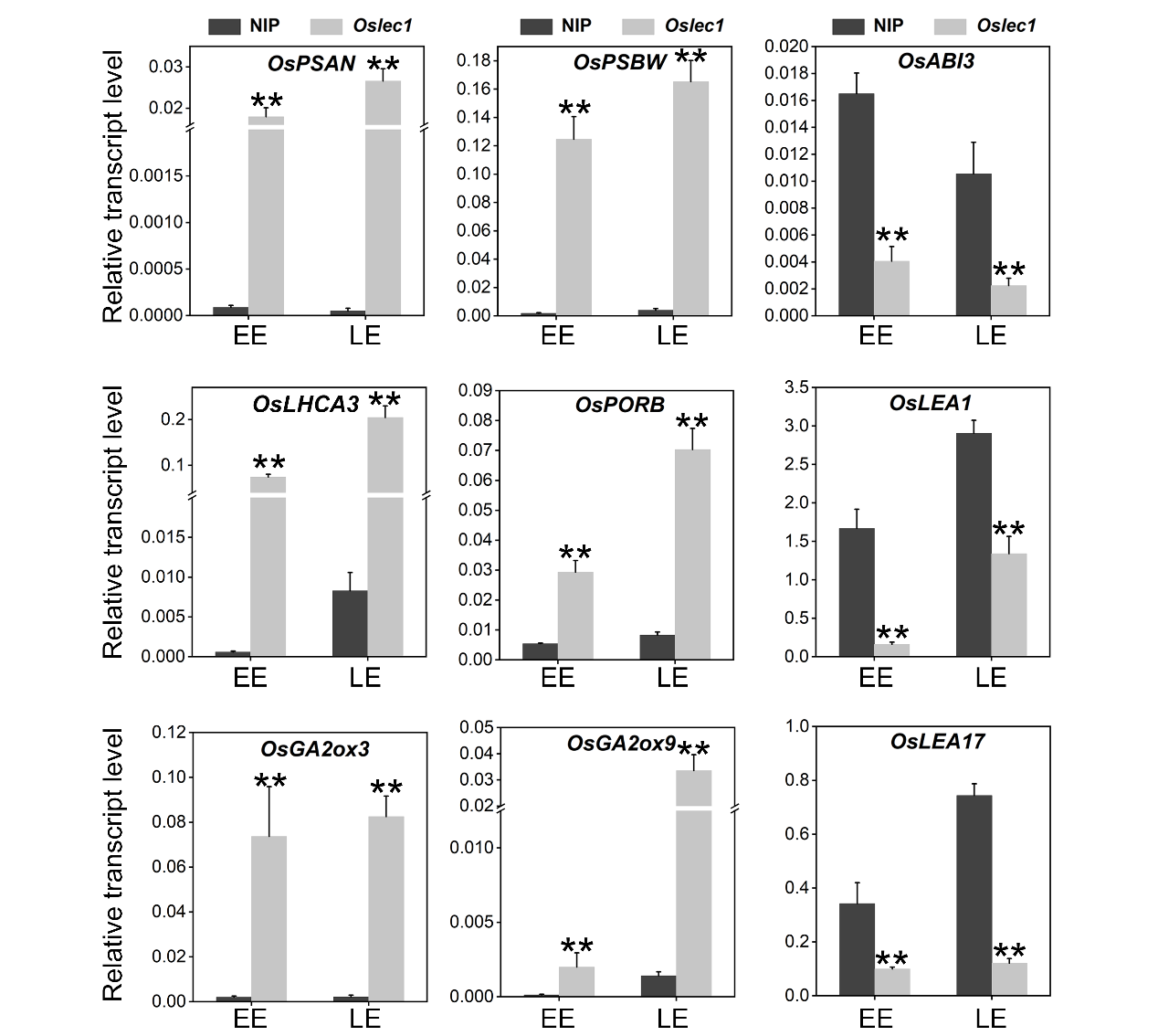


**Fig. S10.** **qRT-PCR analysis of transcription levels of OsLEC1-regulated genes.**

Three biological replicates were performed, and each replicate was tested with three technical repeats. The results were normalized to the expression of rice *Ubiquitin 5* (*OsUBQ5*).


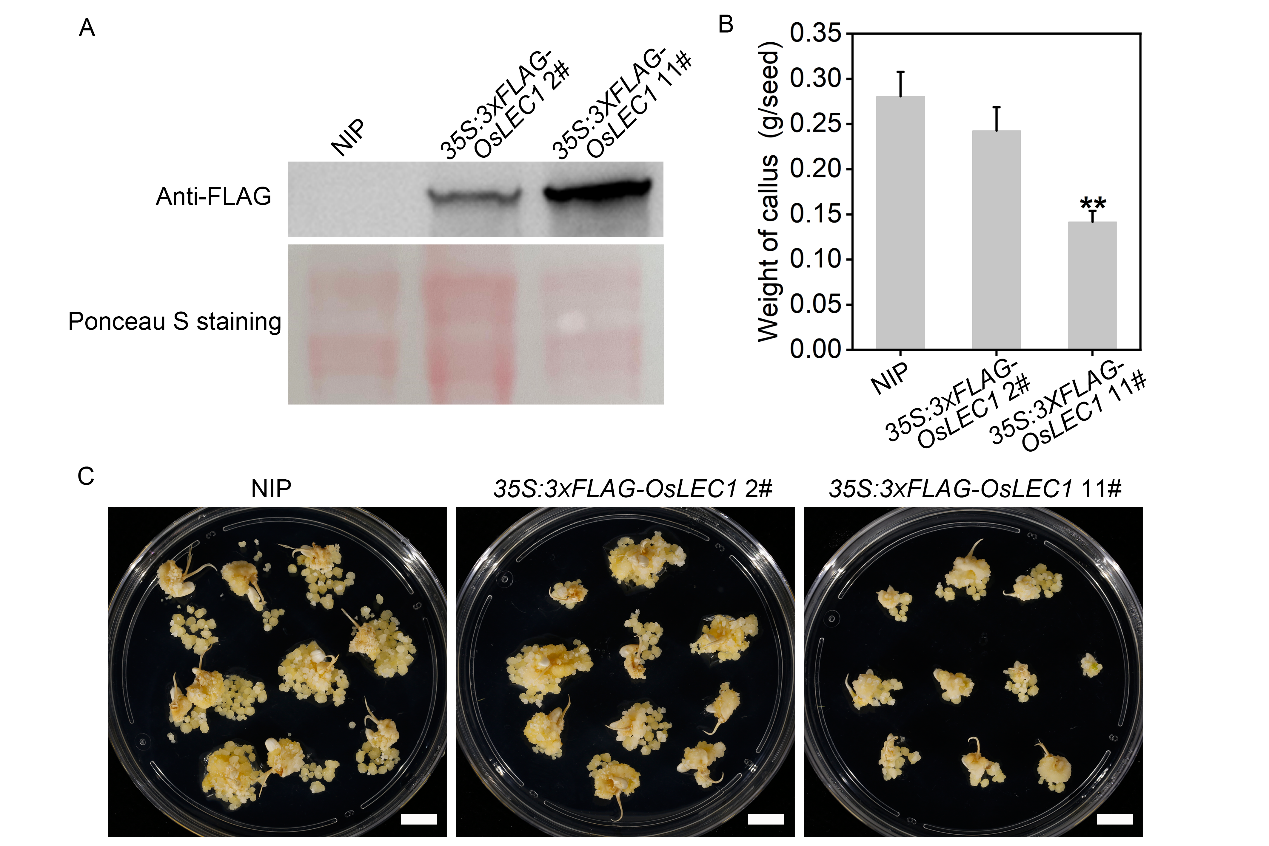


**Fig. S11.Callus formation phenotype of OsLEC1 overexpressed transgenic lines.**

(A) Western blot results of callus of *35S:3xFLAG-OsLEC1* cultured on CIM for 20 days. (B-C) Fresh weight and photos of callus of *35S:3xFLAG-OsLEC1* cultured on CIM for 20 days. Bars, 1 cm.


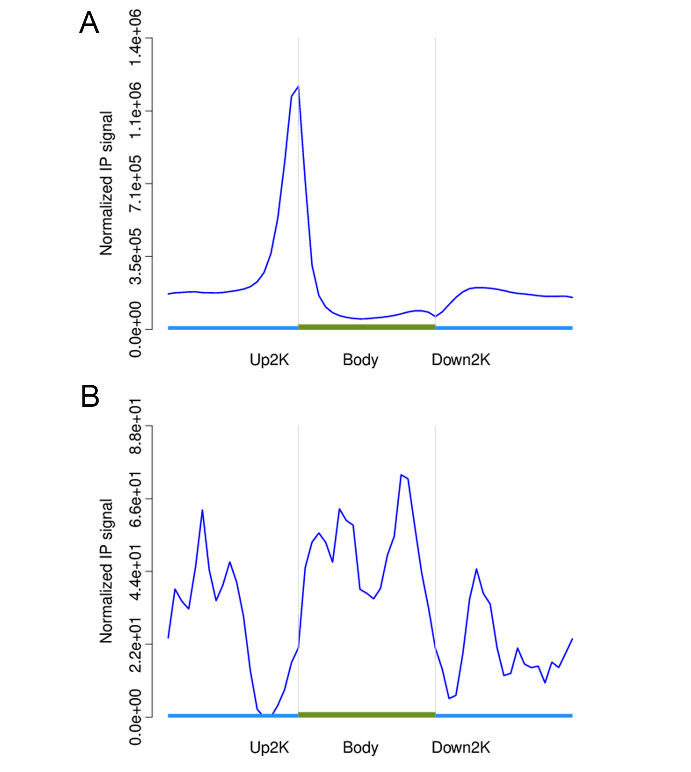


**Fig. S12.** Distribution of reads of ChIP-seq in gene bodies and upstream and downstream sequences. (A) Distribution of reads of ChIP-seq from *35S:3xFLAG-OsLEC1.* (B) Distribution of reads of ChIP-seq from NIP*.*


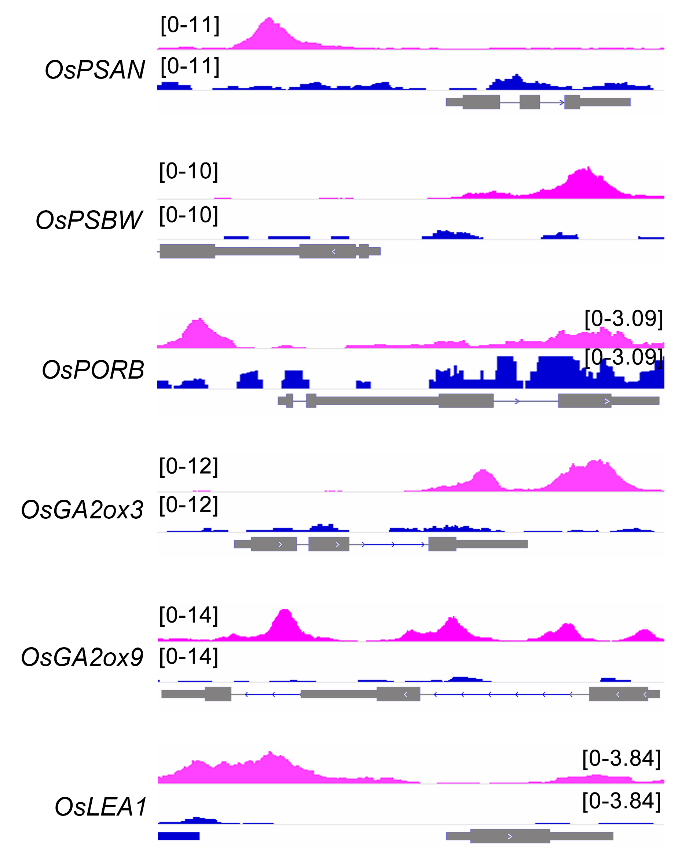


**Fig. S13.** **IGV screenshot of peak sites on genome sequences of OsLEC1-binding genes.** Red color and blue color indicated peak sites of genes in the ChIP-seq results from *35S:3xFLAG-OsLEC1* and NIP, respectively*.* Structure of the target genes were showed in grey color below the peak sites. The number indicate the height range of binding site peaks.


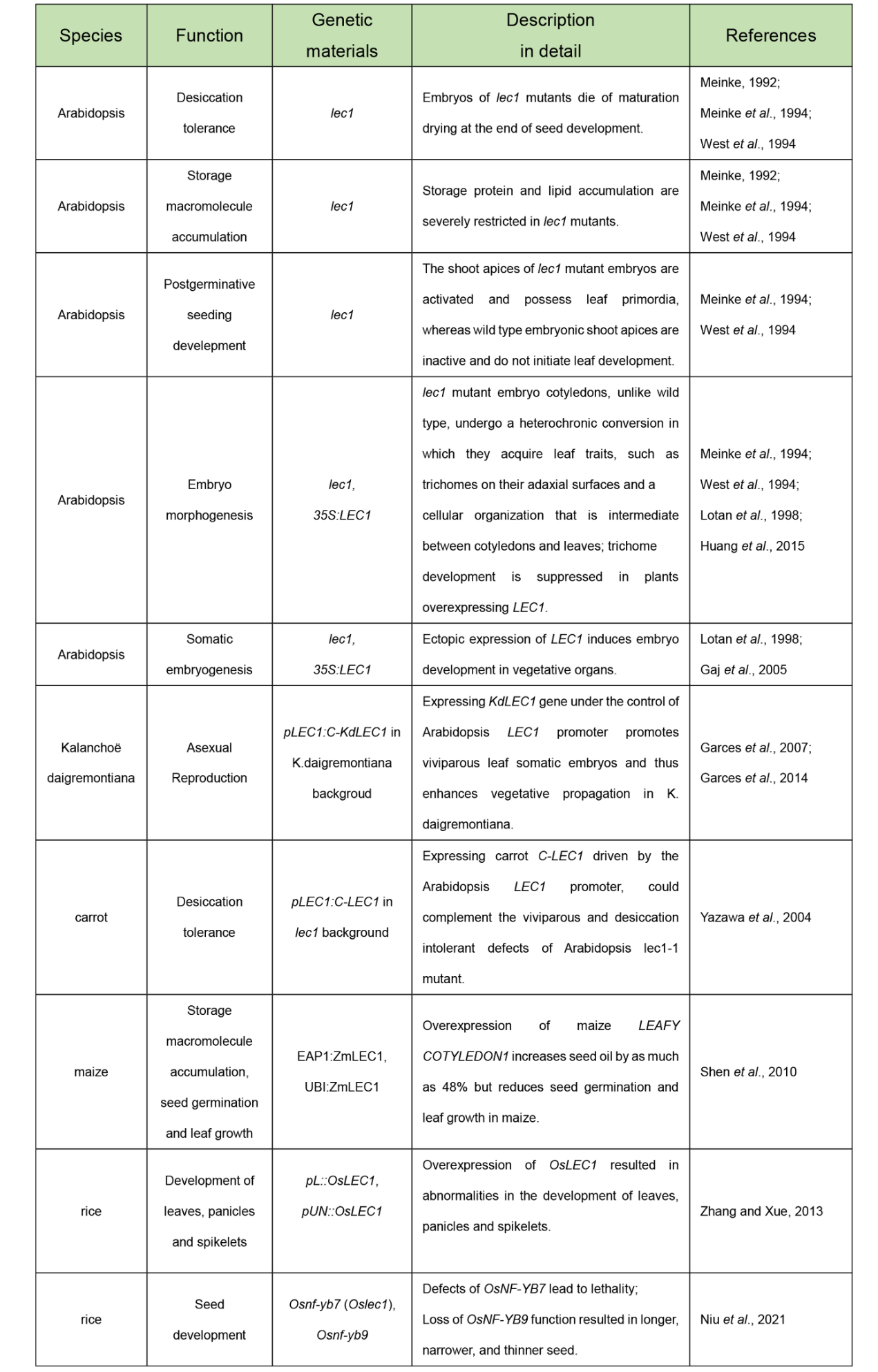


**Fig. S14.** A summary of studies that report the functions of OsLEC1.
